# ^13^C Direct Detected NMR for Challenging Systems

**DOI:** 10.1021/acs.chemrev.1c00871

**Published:** 2022-01-13

**Authors:** Isabella C. Felli, Roberta Pierattelli

**Affiliations:** Department of Chemistry “Ugo Schiff” and Magnetic Resonance Center, University of Florence, Via Luigi Sacconi 6, 50019 Sesto Fiorentino (Florence), Italy

## Abstract

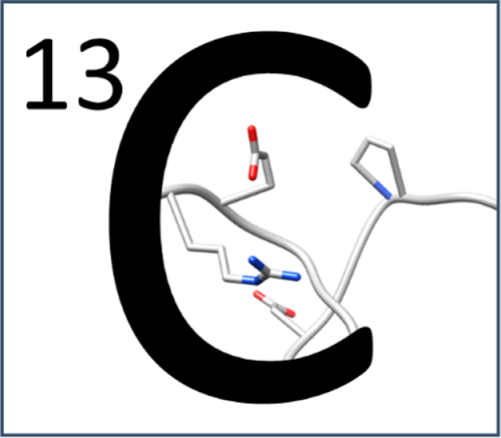

Thanks to recent improvements in NMR spectrometer hardware and pulse sequence design, modern ^13^C NMR has become a useful tool for biomolecular applications. The complete assignment of a protein can be accomplished by using ^13^C detected multinuclear experiments and it can provide unique information relevant for the study of a variety of different biomolecules including paramagnetic proteins and intrinsically disordered proteins. A wide range of NMR observables can be measured, concurring to the structural and dynamic characterization of a protein in isolation, as part of a larger complex, or even inside a living cell. We present the different properties of ^13^C with respect to ^1^H, which provide the rationale for the experiments developed and their application, the technical aspects that need to be faced, and the many experimental variants designed to address different cases. Application areas where these experiments successfully complement proton NMR are also described.

## 1 Introduction

NMR spectroscopy is an indispensable tool for investigations of biological molecules and their interactions. The power of NMR to link structural, dynamic, kinetic, and thermodynamic information makes it an essential component of cutting-edge research in structural biology. Provided NMR spectra can be acquired with high resolution and sensitivity, a virtually unlimited amount of atomic-resolution information can be achieved starting from chemical shift values, nuclear spin relaxation rates, scalar couplings, exchange effects, diffusion coefficients, and other highly sophisticated observables. This places NMR in a unique position with respect to the many spectroscopic methods that provide global information or to different high-resolution methods, such as X-ray crystallography and cryo-electron microscopy, that however fail to provide information about macromolecules in solution for highly dynamic or heterogeneous ones in particular.

Needless to say that continuous development of the experimental approach is necessary to exploit at best this powerful spectroscopic technique to extend the complexity of the systems under investigation, as required by the many challenges in biomedical research. These would benefit from the availability of high resolution structural and dynamic details on biologically relevant molecular components. This in turn triggers instrumental technological improvements that enable expansion of the applications to problems of increasing complexity.

We would like to focus here on the example of carbon-13 direct detection NMR in solution, one of the widely used tools to characterize biological macromolecules. We are going to start by introducing the key properties of heteronuclear spins that make heteronuclear direct detection interesting and discuss the many strategies developed to overcome potential critical points, such as the problem posed by homonuclear decoupling in the direct acquisition dimension. The issue of the starting polarization source as well as coherence transfer methods is described since they constitute common basic ingredients of more complex NMR experiments. The flow of the review then proceeds to illustrate the many experiments developed, focusing on the applications where they reveal unique additional information with respect to more conventional approaches. Current challenging research areas where these methods provide useful data are also presented.

### 1.1 Properties of Heteronuclear Spins

The term “heteronuclear” refers to nuclear spins other than protons, the first ones to be considered in general in NMR spectroscopy (the magnetic field of an instrument, B_0_, is indeed often indicated through the ^1^H Larmor frequency). The most widely used nuclear spins for biomolecular NMR investigations are ^1^H, ^13^C, ^15^N, and ^31^P (the latter of interest for the investigation of nucleic acids); we will focus here on the contributions of ^13^C and ^15^N to the study of proteins even if similar arguments can of course be extended to nucleic acids (including ^31^P) and carbohydrates.

While sharing the same spin angular momentum (*S* = 1/2), heteronuclei have smaller gyromagnetic ratio (in absolute value) with respect to proton and thus smaller magnetic moment. The latter, which interact with the external magnetic field B_0_ determining the Larmor frequency, is responsible for the intrinsic sensitivity that scales down moving from ^1^H to ^13^C to ^15^N.^1^ The magnetic moment associated with nuclear spins is also responsible for the dipole–dipole (DD) interactions with other nuclear spins in the surroundings, interactions that in isotropic solution are largely averaged by fast molecular tumbling and do not have an impact on line positions or do not cause line splitting like the scalar coupling. They are however the major interactions that promote nuclear relaxation. Therefore, in principle heteronuclear spins sense lower dipolar contributions to relaxation as a result of their lower magnetic moments. Considering the interaction between two dipoles at a specified distance, heterouclei sense a lower contribution to relaxation with respect to protons by a factor of approximately (γ_X_/γ_H_)^2^ (neglecting contributions from the different spectral densities). Paramagnetic contributions to nuclear relaxation provide a clear example in this respect. Once a particular paramagnetic center is defined, dipolar contributions to relaxation of nuclear spins in the surroundings depend on the distance (1/*r*^6^) and on the square of the gyromagnetic ratio (γ_X_^2^) of the nucleus itself (as well as on spectral densities). Therefore, at a fixed distance from the paramagnetic center, heteronuclear spins sense a smaller dipolar interaction and thus a smaller contribution to relaxation with respect to protons; in other words, the so-called paramagnetic relaxation enhancements at a specified distance from a paramagnetic center are smaller for heteronuclei than for protons.

Similar arguments hold for diamagnetic contributions to relaxation: dipolar interactions involving heteronuclear spins are lower than those involving protons, not easy to generalize in this case because the final result depends on many contributions which in turn depend on the local chemical structure. On the other hand, contributions to dipolar relaxation deriving from proton spins are generally dominant, both for proton itself and for heteronuclei due to the large γ_H_, a situation that pushed the development of isotopic enrichment in deuterium to reduce the bath of proton dipoles in which heteronuclei are immersed and thus reduce contributions to nuclear relaxation.^2−4^ Another example in this respect is provided by ^15^N relaxation often measured to investigate local dynamics in proteins. In this case, the dominant dipolar contribution to ^15^N relaxation is provided by the directly bound proton.

The other striking difference when moving from protons to heteronuclei consists of the electronic structure/chemical environment that influences signals chemical shift. Largely averaged in solution due to fast molecular tumbling, the isotropic part of the chemical shift tensor determines peak positions. The major contributions to chemical shifts derive thus from the local molecular topology that translates into the different chemical shifts expected for the different functional groups, a property often exploited in the study of small molecules. In proteins, the chemical structure of the different amino acids as well as their link through the peptide bond is the major determinant of the observed signals chemical shift. The local 3D structure provides an additional contribution to it, which clearly shows a general trend toward larger chemical shift dispersion for heteronuclei with respect to protons, as can be appreciated by inspecting the Biological Magnetic Resonance Data Bank (BMRB, https://bmrb.io/), the database in which chemical shifts of assigned proteins are deposited.

The anisotropic part of the chemical shift tensor instead contributes to nuclear relaxation. Therefore, large contributions to relaxation are expected when significant chemical shift anisotropy (CSA) is present such as for example for nuclear spins involved in the peptide bond or aromatic rings, certainly an important aspect to consider when exploiting heteronuclear spins. Several solutions have indeed been proposed to use constructively interference between CSA and DD to mitigate the contributions to transverse relaxation that may broaden lines beyond detection for globular proteins of increasing molecular mass due to the increasing rotational correlation time. On the other hand, these contributions to nuclear relaxation do not have a detrimental impact when focusing on highly flexible proteins, in which the fast motions reduce contributions to transverse relaxation.

Scalar couplings have a strong impact on the spectra, in particular in the direct acquisition dimension, and their magnitude and topology are also influenced by the type of nuclei under investigation. These are mediated by electronic effects and depend on many factors including the gyromagnetic ratio of the nuclear spins involved in the coupling as well as the electronic structure and the local geometry. However, the most striking difference moving from ^1^H to ^13^C relates to the fact that large one-bond homonuclear scalar couplings are present when focusing on ^13^C nuclear spins in uniformly labeled samples, while analogous ones are not observed for protons. This property, widely exploited in many multidimensional experiments to achieve coherence transfer, also causes very large signals splitting and the relative complex multiplet structures complicate direct detection of ^13^C. This constitutes an important aspect to be considered in order to convert ^13^C direct detection into a useful tool for the study of complex macromolecules. On the other hand, when moving to multiple bond effects, these are smaller than the analogous ones involving ^1^H (for example, ranges for ^3^*J*_HH_ are often larger than those for ^3^*J*_CC_ in aliphatic chains).

Heteronuclei constitute the molecular backbone; protons are at the edge of chains of chemical bonds and in many cases form the exposed surfaces of macromolecules. This property is often used in the study of interactions in which changes in ^1^H chemical shifts are investigated to identify interaction surfaces. Also heteronuclei are of course affected by changes in the nucleus surrounding but are more sensitive to changes in the local structure, such as changes in dihedral angles, and these complementary features can result useful.

Proteins are studied in water, the solvent of life. Interactions of proteins with water are key for many aspects: they prompt polypeptide chains to fold into stable globules or to remain flexible and solvent-exposed as well as to create membrane-less organelles through liquid–liquid phase separations. Therefore, the interactions with the solvent have a very relevant role in protein function. Proton NMR can be used to detect changes with pH or protonation state of a protein, but it is also influenced by exchange processes that, depending on their magnitude, can broaden lines beyond detection. Heteronuclei in this context can act as “spies” of nonprotonated states providing information also in cases in which protons are not present or when fast exchange between the free and bound forms broadens ^1^H NMR lines beyond detection.

### 1.2 Building Blocks of NMR Experiments: What’s New for ^13^C Direct Detection?

Heteronuclear spins have interesting properties as also exploited in the indirect dimensions of many multidimensional NMR experiments based on ^1^H direct detection (“inverse detection” of heteronuclei). We would like to discuss here the most important aspects to consider when moving to ^13^C detection.

Starting from simple 1D ^13^C NMR spectra, often used also for the study of small molecules to identify specific functional groups or coupling topologies, the large chemical shifts dispersion is accompanied, when studying isotopically labeled macromolecules, by the onset of complicated multiplets determined by the large one bond homonuclear ^13^C–^13^C scalar couplings. These can range from simple doublets for ^13^C nuclei that only have one ^13^C bound, or to more complicated multiplet structures observed for the different side-chains with ^13^C nuclei directly bound to two or three other ^13^C nuclei. The onset of complicated multiplets, that on one hand can provide valuable information regarding the type of spin system, is detrimental when seeking high-resolution information needed to study macromolecules. Indeed, the complex multiplet structure of ^13^C signals deriving from homonuclear scalar couplings drastically reduces both sensitivity and resolution of the NMR spectra, two key features for the study of complex macromolecules.

Several strategies have become widely used to suppress these homonuclear couplings in the indirect dimensions of NMR spectra such as the inclusion of constant time evolution periods, the use of band-selective inversion pulses to refocus scalar coupling evolution, etc.^5^ In many cases, the resolution that can be achieved in the indirect dimension is however limited by the number of points that can be acquired in the time that can be dedicated to a specific experiment and the reduction in resolution brought about by signals’ splitting is seldom a limiting factor. On the other hand, when nuclear spins are investigated in the direct acquisition dimension, the FID can in principle be acquired as long as desired (just limited by the transverse relaxation properties of the system under investigation rather than by the time needed to accumulate increments to construct an indirect dimension). This constitutes a contribution to spectral resolution, provided the complex multiplet structures are simplified by the implementation of homonuclear decoupling. The problem of ^13^C homonuclear decoupling is more demanding than heteronuclear decoupling because the two nuclear spins involved in the coupling are close in frequency and thus radio frequency irradiation on one of them can be sensed by the other. However, several elegant solutions have been proposed and constitute a relevant aspect for the design of NMR experiments for the study of complex biomolecules.

#### 1.2.1 Homonuclear Decoupling

Let us start discussing the “simple” case of a ^13^C nuclear spin that only has one large bond homonuclear coupling such as for example carbonyl nuclear spins (^13^C′) in protein backbones, which share a large one bond scalar coupling with ^13^C^α^ nuclear spins. One nice feature of the one bond scalar couplings (^1^*J*_C′C^α^_) consists of the fact that they are not as variable as, for example, three bond couplings, which largely depend on the local conformation of the molecule. In the case of carbonyl moieties of protein backbone, the one bond scalar coupling with C^α^ is fairly uniform throughout the primary sequence and relatively independent of the type of amino acid and on the local conformation,^6^ a property that renders the problem of homonuclear decoupling generally amenable and more easy to address with respect to, for example, ^1^H homonuclear decoupling. The most straightforward approach thus consists of deconvolution of the spectra, exploiting a defined value of the coupling. Initially proposed for indirect dimensions of triple resonance experiments^7^ and implemented for ^13^C detected experiments,^8,9^ this approach has been recently revived by the incorporation of AI methods.^10^

**Figure 1:**
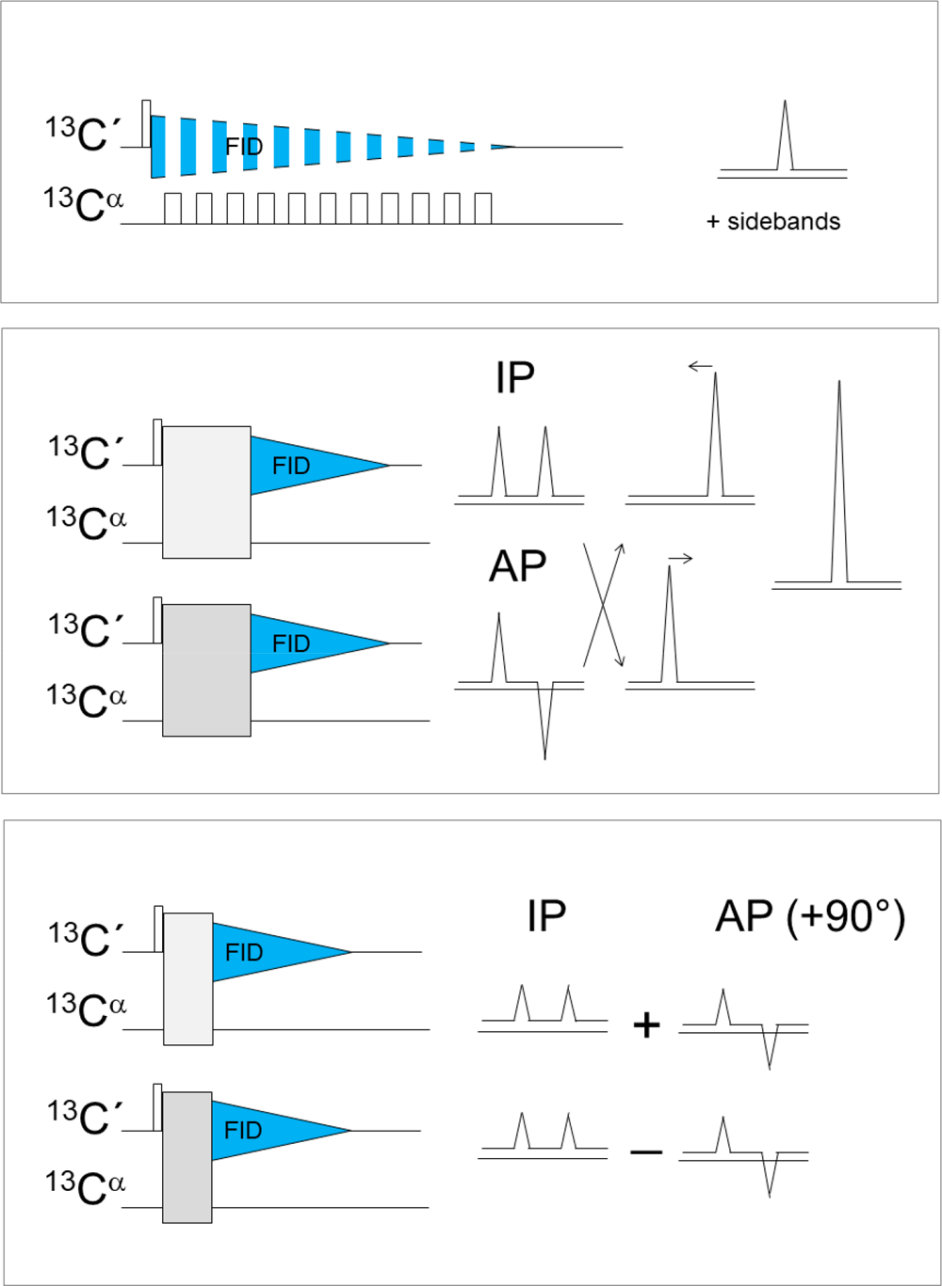
Homonuclear decoupling strategies initially implemented for ^13^C direct detection: band-selective homonuclear decoupling (top panel), virtual decoupling achieved through the IPAP (middle panel), and S^3^E (bottom panel) approaches. A scheme illustrating the linear combinations performed to achieve virtual decoupling is also reported on the right side of the middle panel. The contribution of the IP and AP components to the two independent FIDs acquired through the S^3^E approach is also schematically indicated on the right side of the bottom panel.

The other possibility, provided the chemical shifts of the two nuclear spins involved are sufficiently different to allow for their selective irradiation, consists of band-selective homonuclear decoupling in which the acquisition time is shared between acquisition and decoupling mode in alternating time intervals (Figure 1).^11−13^ This approach requires an additional radio frequency channel; decoupling sidebands are observed depending on the frequency of acquisition and decoupling periods. An elegant alternative approach to homonucear decoupling in which the acquisition time is shared between acquisition and 180° refocusing pulses is the BASHD.^14^ Introduced for solid-state NMR experiments,^15^ it was adapted for direct detection of carbonyl carbon nuclei in solution measurement.

The most widespread approach used nowadays consists of using virtual homodecoupling, that is exploiting spin-state-selective methods in which scalar couplings are preserved and different components of the signal are collected: these constitute the basis to achieve virtual decoupling through the appropriate linear combinations of the acquired signals to separate the different multiplet components, followed by a shift to the center of the original splitted signal.^16^

Several experimental variants based on this idea have been developed and may result useful for different applications. The most straightforward implementation of this idea consists of the IPAP approach^17^ in which the in-phase (IP) and antiphase (AP) components of the carbonyl carbon signals (with respect to C^α^) are acquired and separately stored (Figure 1).^18−20^ The postacquisition treatment of the acquired data, which can also be performed directly in the time domain, enables the removal of the large one bond splitting from the spectra. Interestingly, this approach preserves the coupling and can in principle be applied to mutually decouple and still observe the two nuclear spins involved in the coupling, as demonstrated in solid-state applications.^21^ An experimental variant in which AP and IP signals for virtual decoupling are acquired sequentially in a single scan has been recently proposed.^22^

When fast transverse relaxation becomes a limiting factor, shorter experimental variants can be implemented.^18,20,23^ Indeed the IPAP approach relies on complete interconversion between the IP and AP components of the signal and requires a time that is inversely proportional to the scalar coupling itself (1/2J_C′C^α^_). However, partial interconversion between in-phase and antiphase already provides the two components in half the time; in this case, changing the sign of one of the two components in alternate scans allows storage of two FIDs that can be used to separate the two multiplet components needed to perform homonuclear decoupling through appropriate manipulations of the acquired FIDs (Figure 1).^20^

Direct acquisition of the AP signal component was also proposed for systems in which transverse relaxation is a key limitation such as for very large proteins^24^ or for paramagnetic ones.^25^ Finally, different variants exploiting sensitivity enhancement strategies were proposed for homonuclear decoupling in COCA (COCAINE)^26^ and CON^27^ experiments. They were also shown to be useful alternatives for heteronuclear decoupling.^28^

These principles implemented for backbone carbonyl homonuclear decoupling can of course be extended to analogous cases such as ^13^C nuclear spins that only have one large scalar coupling with a second ^13^C nuclear spin that also has a different chemical shift, sufficiently different to allow band-selective inversion of the two spins independently. Indeed this strategy also performs well for amino acid side-chains that have a carbonyl/carboxylate moiety such as aspartate, glutamate, asparagine and glutamine residues. It has also been implemented to investigate terminal nuclear spins of aliphatic side-chains and to decouple them from their next neighbors.^29−32^

The situation becomes more complex for spins that are coupled to more than one additional ^13^C spin through large one-bond scalar couplings such as ^13^C^α^ spins (for all amino acids except glycine) as well as for the vast majority of spins of amino acid side-chains. The ideas described can in principle be extended also to this case; selected applications were so far implemented. As an example, several approaches were proposed to perform homonuclear decoupling of ^13^C^α^ signals by clever combinations of spin-state selective approaches (DIPAP, DS^3^E).^20,30^ The capacity to selectively invert ^13^C^α^ from ^13^C^β^ (in addition to ^13^C′) constitutes a key feature, and some compromises may be necessary for signals with similar ^13^C^α^ and ^13^C^β^ shifts that fall close to the transition regions of the inversion profiles of the band-selective pulses employed. The acquisition of the different components also has a cost in terms of overall sensitivity. Despite these additional complications, these methods were successfully used to investigate very large proteins in which extensive isotopic enrichment was mandatory and for which carbonyl carbon direct detection provided too broad lines.^30^ Similar approaches were also proposed for the investigation of nucleic acids through ^13^C direct detection^33−37^ as well as to focus on aromatic residues in proteins.^38^

#### 1.2.2 Starting Polarization Source

The starting polarization source used in NMR experiments also constitutes an important element that can be used to modulate experimental sensitivity. On the other hand, the latter also depends on the kind of information desired. Direct detection of heteronuclei for biomolecular applications in solution, after pioneering work in 1988^39^ was abandoned in favor of inverse detection methods.^40−42^ It was proposed at the beginning of 2000 as a strategy to recover information on paramagnetic proteins in regions in which proton resonances are broadened beyond detection due to paramagnetic relaxation enhancements.^38,43−47^ Dipolar contributions to relaxation sensed by nuclear spins when a paramagnetic center is present in a molecule depend on the properties of the paramagnetic center itself, on the effective correlation time modulating the interaction, on the electron–nuclear distance (1/*r*^6^), and on the square of the gyromagnetic ratio of the nuclear spin under investigation (γ_X_^2^).^48^ Therefore, shifting our attention from ^1^H to ^13^C and ^15^N ensures reduction of the paramagnetic enhancement (considering the same distance); from a different point of view, similar enhancements are sensed at shorter distances from the paramagnetic center and thus shifting the focus from ^1^H to ^13^C (and to ^15^N) enables researchers to observe resonances of nuclear spins closer to the paramagnetic center since the additional contributions to relaxation are scaled by (γ_X_/γ_H_)^2^.^49,50^

When considering experiments that are more complicated than 1D experiments, these should not actively perturb ^1^H nuclear spins in any of the coherence transfer steps because this would reintroduce the dependence on ^1^H transverse relaxation, much more sensitive to paramagnetic relaxation enhancements. For this reason initial variants of ^13^C detected experiments for biomolecular applications in solution were based on ^13^C as a starting polarization source and never exploited protons in any of the coherence transfer steps; these experiments were called “protonless” NMR experiments.^20^ These experiments also resulted useful for the study of very large proteins in which high levels of deuteration were necessary^51^ reducing the amount of information that could be achieved through proton direct detection.^9,19,52^

While focusing on this topic, it became evident that heteronuclear direct detection could result useful also for different applications in which ^1^H fast relaxation does not constitute a limiting factor.^23,53,54^ For these applications, the use of ^1^H as a starting polarization source brings a significant increase in experimental sensitivity,^55−58^ still exploiting heteronuclear chemical shifts in all the detected dimensions of multidimensional NMR experiments to benefit from their contribution to resolution, a key feature when focusing, for example, on proteins devoid of a stable tertiary structure. These experiments were thus generally referred to as “exclusively heteronuclear” to indicate that only heteronuclei were frequency labeled in all dimensions, regardless of the starting polarization source exploited.^24,28,53,59^

**Figure 2:**
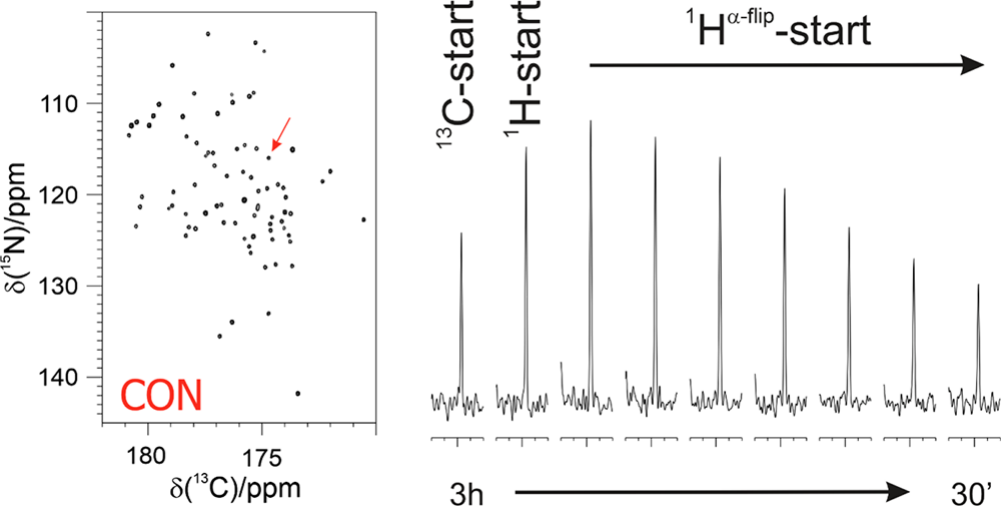
Intensity of one of the cross peaks obtained in 2D CON experiments (the correlation for C′59–N60 of ubiquitin indicated by an arrow in the full spectrum reported on the left) recorded with different pulse schemes and different interscan delays (d1) is compared by showing its trace. From left to right: ^13^C-start (relaxation delay d1 = 2.5 s), ^1^H-start (d1 = 1.5 s), and ^1^H^α-flip^ with different d1 (1.5, 1.2, 0.9, 0.7, 0.5, 0.35, 0.2 s). Adapted from ref (59). Copyright 2009 American Chemical Society.

Considering for the moment backbone nuclear spins, the proton polarization source can be provided by amide protons (^1^H^N^) as well as by aliphatic protons (^1^H^α^) with advantages and disadvantages that depend on the properties of these two nuclei (Figure 2). Amide protons, which can be easily correlated to the directly bound nitrogen and then to carbonyl carbon nuclei through the ^1^*J*_HN_ and ^1^*J*_C′N_, respectively, are influenced by solvent exchange that can become so pronounced that coherence transfer becomes inefficient. In addition proline residues do not have an amide proton, a feature that reduces the information content of CON spectra that exploit ^1^H^N^ polarization as a starting source, in particular for proline-rich proteins.

Backbone aliphatic protons (H^α^) are instead present for all amino acids and are nonexchangeable nuclei. Therefore, experiments based on ^1^H^α^ as a starting polarization source can in principle provide complete information. Also in this case coherence transfer pathways exploiting large scalar couplings (^1^*J*_H^α^C^α^_, ^1^*J*_C^α^C′_, and ^1^*J*_C′N_) enable the transfer of ^1^H polarization to backbone nuclear spins in an efficient way without major losses; care should be taken to consider the role of ^13^C^α^–^13^C^β^ couplings in the coherence transfer pathway to ensure that no information is lost for amino acids with similar ^13^C^α^ and ^13^C^β^ chemical shifts.

Longitudinal relaxation enhancement (LRE) strategies can be used to reduce the interscan delay and reduce experimental time (or increase the S/N per unit time).^60^ Borrowing ideas proposed for amide proton detected NMR experiments, in which amide protons are selectively perturbed to enhance the recovery to equilibrium and reduce interscan delays (SOFAST, BEST),^61,62^ different variants of ^1^H^N^-start experiments were proposed exploiting ^1^H^N^ band-selective pulses (H^NBEST^CON).^63^ It is interesting to note that in these experiments ^1^H polarization is only used as a starting polarization source so that the longitudinal recovery starts well before acquisition of the FID, a feature that enabled the acquisition of the H^NBEST^CON without introducing any longitudinal recovery delay after the acquisition of the FID.^63^

Similar longitudinal relaxation enhancement approaches would be useful also for ^1^H^α^ protons, used as a starting polarization source in several variants of exclusively heteronuclear NMR experiments. However, ^1^H^α^ spins are more difficult to manipulate through band-selective pulses with respect to the case of ^1^H^N^ protons because they fall in a more crowded spectral region. To this end, a variant to selectively manipulate a subset of nuclear spins while leaving others unaffected was proposed that exploits the scalar coupling with the attached heteronuclear spins (H^flip^).^59^ In this way, longitudinal relaxation enhancement can be implemented in a general way for protons used as a starting polarization source of exclusively heteronuclear NMR experiments, both for ^1^H^N^-start and ^1^H^α^-start variants (Figure 2).

The use of proton–nitrogen cross-polarization, in place of the more widespread INEPT block, was also proposed since this excitation mechanism uses the large water-magnetization reservoir to continuously replenish the amide-proton; combined with the CON reading scheme, this represents another useful approach to study systems in which solvent exchange is very pronounced.^64,65^

## 2 Suite of ^13^C Direct Detection NMR Experiments

### 2.1 Sequence-Specific Assignment

Similarly to ^1^H detected experiments, ^13^C detected experiments can be differentiated based on the active interactions exploited in the coherence transfer steps (scalar or dipolar coupling), the starting polarization source (^13^C or ^1^H), and the kind of detected nuclei (^13^C′ or ^13^C^α^ or others).

The experiments initially proposed for assignment purposes were based on ^13^C-start, ^13^C-detection, and completely avoided protons in any of the magnetization transfer steps (protonless NMR experiments).^20^ The set of protonless NMR experiments included various kinds of 2D experiments correlating carbon nuclei such as the ^13^C–^13^C COSY, ^13^C–^13^C TOCSY, and the CACO MQ correlation experiment.^20,30,43,45,46,66,67^ The ^13^C–^13^C NOESY experiment proved also useful in cases in which fast transverse relaxation represents a major limitation.^9,19^ Despite the ^13^C–^13^C cross-relaxation rates being expected to be much smaller than ^1^H–^1^H ones, the ^13^C–^13^C NOE effects between directly bound carbon nuclei are easily detectable and spin diffusion through their nuclei is a very efficient process able to provide very useful spectra for amino acid type identification. These initial results opened the way to the development of a wide variety of experiments for sequence-specific assignment.

A backbone ^13^C nucleus that can be used to design a whole series of experiments is C^α^. It is characterized by a large chemical shift dispersion, so it can provide highly dispersed spectra. However, the coupling to the C^β^ present in all amino acids other than glycine makes homonuclear decoupling less straightforward (see section 1.2.1). Nevertheless, the CAN experiment is useful to detect the two correlations between C^α^ and the intra- and inter-residue nitrogen.^50^ Variants to highlight the sequential correlation and thus discriminate it from the intraresidue have been proposed.^68^ These gain also in resolution since the inter-residue correlations are generally more resolved than the intraresidue ones.^69^

Thanks to the potential of experiments based on C^α^ direct detection for the study of higher molecular mass proteins, it was proposed to exploit the isotopic labeling strategy designed for solid-state applications that avoids neighboring ^13^C spins (and thus also the need for homonuclear decoupling in the direct ^13^C dimension).^51,70^ Several experiments based on C^α^ direct detection exploiting this isotopic labeling scheme were proposed including the CANCA and the CACA TOCSY experiments.^68,71^

**Figure 3:**
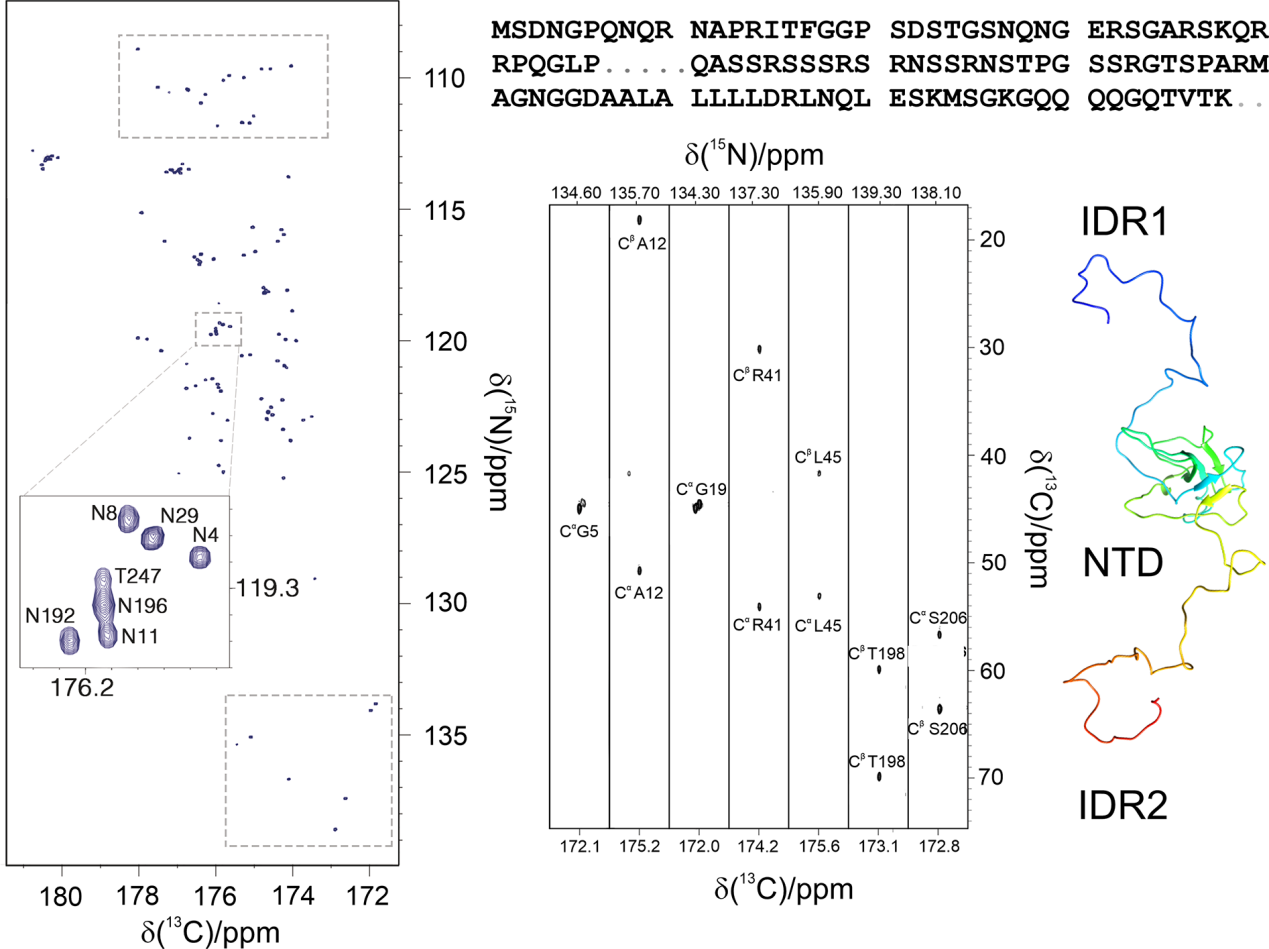
Left: The 2D CON of a 248 amino acids long construct of the SARS-CoV-2 nucleocapsid protein comprising the NTD folded domain and the two intrinsically disordered regions flanking it (IDR1-NTD-IDR2). The high resolution provided by this experiment enables to easily resolve resonances in the usually very crowded Gly-region (upper squared region) and to directly observe connectivities involving proline residues (lower squared region). In the expansion shown in the center of the map, the resolution of several repeating fragments comprising asparagine residues can be appreciated (the assignment reported is referred to the amide nitrogen of the mentioned amino acid). Right: Seven strips derived from the 3D (H)CBCACON experiment extracted at the ^15^N chemical shift of proline residues, flanked by a cartoon of the IDR1-NTD-IDR2 construct. The C′, C^α^, and C^ß^ frequencies belong to the preceding amino acid leading to the X-Pro assignment. The upper part of the panel reports the IDR1-NTD-IDR2 primary sequence (the sequence of the NTD domain is omitted for sake of clarity). Five proline residues are found in the IDR1 and two in IDR2 domain. Adapted from ref (73). Copyright 2021, The Authors under the terms of a Creative Commons CC BY license http://creativecommons.org/licenses/by/4.0/.

The most successful applications of ^13^C direct detected NMR rely on carbonyl carbon detection, which presents a single and uniform splitting that can be easily removed (see section 1.2.1). Carbonyl carbon nuclei can be directly correlated to the neighboring C^α^ nuclei through a ^13^C–^13^C transfer step (^1^*J*_C′C^α^_), which can be further correlated to the C^β^ and to all the aliphatic side chain to obtain the 2D CBCACO and 2D CCCO experiments.^20^ By introducing a further transfer step, it is also possible to include an additional dimension in which the attached nitrogen is frequency labeled.^20,72^ Therefore, the 3D experiments CACON, CBCACON, and CCCON were designed.^72^ These enable the identification of all the spin systems in a protein including those involving proline residues. Actually the correlations involving the proline nitrogen with the carbon frequencies of the previous amino acid (^13^C′, ^13^C^α^, ^13^C^β^, and other aliphatic ^13^C spins) often become the starting point for sequence-specific assignments because, already at this initial stage, they provide inter-residue information regarding X-Pro pairs, as illustrated for two intrinsically disordered regions of the nucleocapsid protein from SARS-CoV-2 (Figure 3).^73^

Several additional experiments were then proposed in which different couplings were exploited to detect the complementary correlations to link amino acid spins systems in a sequence-specific manner. The CANCO experiment,^20,74^ and its variant also including the information about the C^β^ chemical shift (CBCANCO),^75^ provides the complementary information for sequence-specific assignment. These experiments exploit C^α^ and C^β^ chemical shifts to match different spins-systems identified in CBCACON/CCCON spectra and enable researchers to link them to achieve their sequence-specific assignment. However, additional information is useful when focusing on complex systems, in particular when contributions to chemical shifts are drastically reduced due to high flexibility and disorder of the polypeptide. Other experiments were thus designed that provide correlations involving carbonyl carbon nuclei as a further tool to reduce potential ambiguities.^23,67,76^ This is the case of the COCON experiment, which correlates the backbone nitrogen with the attached carbonyl carbon and with the previous and following carbonyl carbon nuclei in the sequence exploiting the ^3^*J*_C′C′_ scalar coupling.^23,76^

As previously described, in most cases of practical interest it is possible to exploit ^1^H as a starting polarization source to boost sensitivity. This has been accomplished for the multinuclear experiments described above to obtain the (H)CBCACON, (H)CCCON, (H)CBCANCO, and (HCA)COCON variants.^28,77^ Moreover, additional experiments were designed that needed a leap in S/N to be feasible due to their intrinsic low sensitivity such as the case of the 3D NMR experiments correlating several residues in a raw ((H)NCANCO, (H)N(COCA)NCO, and (HN)CO(CA)CON).^28,78^

**Figure 4:**
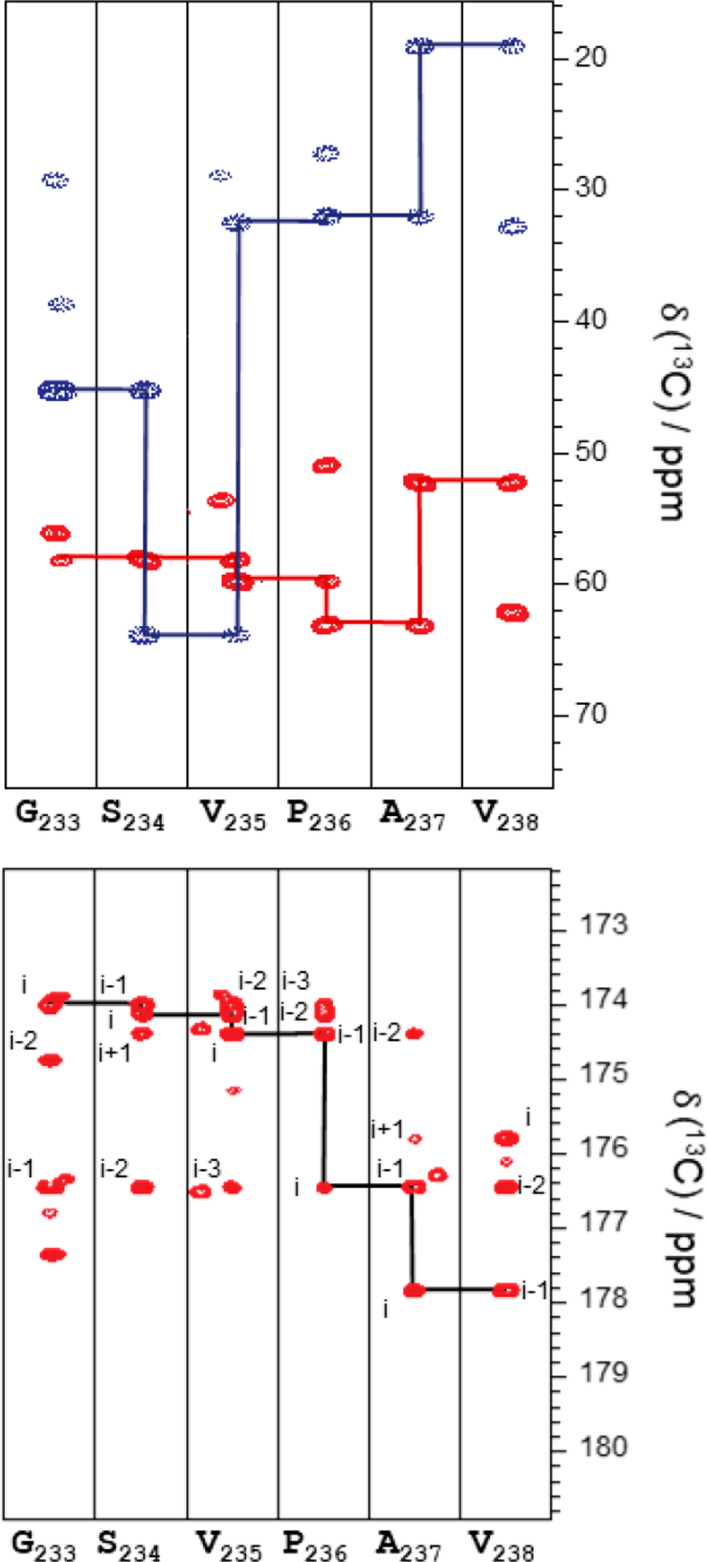
Assignment strips for quail osteopontin. Upper panel: Strip plots of the region 233–238 of the 3D (H)CBCANCO allowing the connectivity of dipeptides by C^α^ and C^β^ carbon nuclei. Lower panel: 3D (HCA)COCON strip plots of region 233–238 showing the connectivity between at least three consecutive C′ signals. Reprinted in part from ref (77). Copyright 2019 Elsevier Ltd. All rights reserved.

As an example of the quality of the spectra, a few slices extracted from the 3D spectra acquired on quail osteopontin, a disordered protein, illustrate the process of sequence-specific assignment through this strategy based on 3D C′-detected NMR experiments (Figure 4).

In the case of particularly crowded spectra, these experiments can also be tailored to select resonances of specific amino acids to simplify the spectra. There are many ways to select the signals of a specific amino acid or a group of amino acids with common characteristics. Implementation of multiple quantum filters to select XH_n_ groups, exploitation of band selective pulses for specific nuclei excitation, tuning of specific delays to select coherence transfer pathways, or matching of increasing numbers of coherence transfer steps to a particular spin topology are all selection blocks that can be included in a pulse sequence. With combinations of these approaches, it is possible to simplify the spectra and identify resonances belonging to virtually all different amino acids.

These experiments are particularly well suited to simplify crowded spectra of highly flexible proteins, which generally have favorable relaxation properties that limit losses resulting from the multiple magnetization transfer steps used for filtering, and for which band-selective pulses are highly effective due to the reduced chemical shifts dispersion.

**Figure 5:**
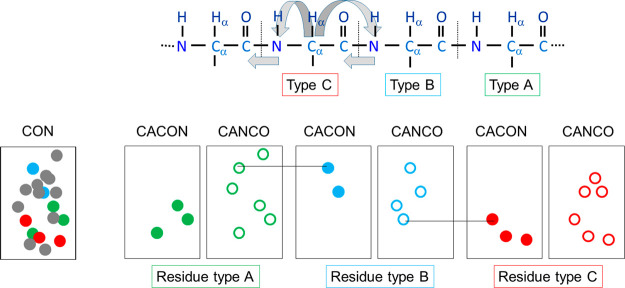
Schematic diagram showing the sequence specific assignment strategy using the amino acid-selection approach for three generic amino acids A, B, and C. The correlations obtained by (CA)CON-based experiments are reported as filled circles, while the additional ones obtained by the (CA)NCO-based experiments are reported as open circles.

The first set of experiments dedicated to this purpose was a ^13^C-adaptation of the MUSIC approach developed by the Oschkinat group.^79−81^ These experiments, based on the CACON and CANCO sequences, are simple in their implementation and can provide information to aid sequential assignment^82^ (Figure 5). On the other hand, many experiments should be acquired to discriminate as much as possible the different amino acids and in some cases the sensitivity is limited by the many transfer steps and pulses included for optimal selection.

A second approach, based on the CBCACO sequence, proposed the use of a selection method based on the ^13^C^β^ topology to distinguish the different amino acid types.^83^ From a single experiment recorded with a sequence that includes several selective pulses cleverly chosen, it is possible to generate subspectra in which the residue signals can be grouped in six classes according to their topology. The classification is coarse but useful for specific purposes.

Although these amino acid selective experiments produce spectra similar to those obtainable by amino acid selective labeling, they offer the clear advantage that only a single sample is required for all experiments and can be exploited in case a specific subset of residues should be monitored, for example, for simplified chemical shift mapping.

### 2.2 Multidimensional Experiments (nD, *n* > 3)

The boost in S/N ratio achieved with pulse sequence design and hardware innovations opened the way to the implementation of experiments with many coherence transfer steps, such as multidimensional experiments capable to correlate many diverse heteronuclei, and, when useful, for assignment purposes, reintroduce also the proton dimension.^84^ These experiments, as for the ^1^H-detected counterparts, require a very long experimental time and thus it becomes crucial to implement approaches to obtain highly resolved spectra in a reasonable time frame.

The acquisition of a multidimensional NMR experiment is generally performed sampling time-points equally spaced on a Cartesian grid (with time-point, we refer to each repetition of the experiment with different delays for chemical shift evolution). This is dictated by the Nyquist theorem, which states that the interval between the time-points sampled cannot exceed the inversion of the spectral width within which all the expected peaks appear. On the other hand, the resolution of the NMR signals is inversely proportional to the length of the acquisition time. This results in an enormous increase of experimental time as the dimensionality of the experiment is increased, especially when a large number of increments is required to achieve optimal resolution in the indirect dimensions.

In the last decades, alternative sampling approaches known with the collective name of nonuniform sampling (NUS) have been introduced in NMR to reduce experimental time, or achieve higher resolution in the same amount of time or be able to acquire spectra of high dimensionality with appropriate resolution.^85^ NUS eludes the limitation of the conventional acquisition scheme by sampling only a subset of the time points of the Cartesian grid, according to some predetermined sampling scheme. The price to pay is that the data cannot be processed with the usual fast Fourier transform (FFT) but need different strategies for proper treatment of the acquired data and for reconstructing the final data-matrix including some postprocessing to remove spectral artifacts in the final spectrum arising from the so-called “sampling noise” intrinsically related to the method.^86^ Several algorithms for data reconstruction or processing have been proposed and optimized such as maximum entropy (ME),^7^ multidimensional decomposition (MDD),^87^ compressed sensing (CS),^88^ and multidimensional Fourier transform (MFT)^89^ to mention just a few. Some of these reconstruction methods have been implemented in the acquisition programs of the spectrometers to be routinely used, not only for ^13^C direct detected experiments of course.

The use of NUS in ^13^C direct detected spectra was tested first with the CANCO experiment on ubiquitin, a small globular protein of 73 amino acids.^59^ Even without the inclusion of ^1^H as starting polarization source, it was possible to reduce at about 40% the number of acquired points, reconstructing the final data matrix with the MDD algorithm.^59^ Different approaches were also tested for the 3D CBCACON experiment.^90^

In the following years, a number of additional experiments with higher dimensionality were designed, all tailored for spin system identification and backbone resonance assignment. The most successful example is provided by the suite of experiments developed by Koźmiński and co-workers, which takes advantage of the sparse multidimensional Fourier transform (SMFT) method to handle the spectra.^91^ One of the advantages of this method is the possibility to process only a subspace of the full multidimensional spectrum at arbitrary frequency coordinates and to simplify the analysis of multidimensional spectra by displaying only 2D cross-sections computed at some predefined frequencies collected in a lower dimensionality “basis spectrum” (2D/3D), which shares the same dimensions with the higher dimensionality experiment (4D/5D). In this way, the inspection of the 4D/5D is reduced to the analysis of a collection of lower dimensionality maps. The complete set of experiments, particularly useful for the investigation of intrinsically disordered proteins, has the CON and the CACON experiments as reference spectra to process 4D and 5D data sets, respectively. The choice of these two is based on the fact that they provide the best result in terms of reliability of the sequential backbone assignment taking advantage of the excellent chemical shift dispersion of the resulting spectra.^92,93^

The multidimensional ^13^C detected NMR experiments that have been proposed share as common features direct detection of carbonyl carbon nuclei and exploit coherence transfer pathways mediated by the one-bond and two-bonds scalar couplings between backbone heteronuclear spins (^1^*J*_C′N_, ^1^*J*_C^α^N_, ^2^*J*_C^α^N_, ^1^*J*_C^α^C′_). Since these involve coherence transfer between several nuclear spins, the extension to higher dimensions can be easily achieved through frequency labeling of different spins exploited for coherence transfer. The most straightforward approach thus consists of extending the dimensionality of 3D experiments by frequency labeling additional nuclear spins exploited in coherence transfer pathways, as proposed for 4D HCBCACON, 4D HCCCON, 4D HCBCANCO, 4/5D HNCACON and 4/5D HNCANCO, and 3/4D HCANCACO experiments.^94,95^ Additional variants were also proposed that exploit the most informative and well-resolved heteronuclear spins in the indirect dimensions, such as the 5D CACONCACO to focus on backbone assignment, followed by the 5D HC(CC-TOCSY)CACON^96,97^ to discriminate between different amino acid types.

An approach to accelerating the long NMR experiments consists of the implementation of ^1^H-start and longitudinal relaxation enhancement that significantly shortens the effective longitudinal recovery time and allows for shorter delays between the consecutive acquisitions (section 1.2.2). This was exploited to propose improved variants of the 4D and 5D NMR experiments based on selective excitation of ^1^H^N^ nuclei (not touching the aliphatic ones as well as the water resonance),^96^ or on the ^1^H^flip^ approach that can be exploited also when ^1^H^α^ are used as a starting polarization source.^92^ Several variants of the same core NMR experiment can be used (^1^H^NBEST^, ^1^H^N-flip^, ^1^H^α-flip^) allowing researchers to perform a sequence specific walk through the backbone by “hopping” from one CON correlation to the neighboring one (CON-CON strategy). These experiments can be performed as 4D experiments (by using a 2D CON as a reference) as well as in the 5D version in which the reference spectrum is the 3D CACON. The proficiency of this CON-CON strategy then led to the design of the analogous experiments based on ^1^H direct detection,^98^ providing a complete set to meet different experimental needs.^99−102^ Importantly, this set of spectra is well suited for the implementation of automated protocols for assignment.^103,104^

Another strategy that can be used to speed up the acquisition of a multidimensional spectrum is to exploit projection spectroscopy, in which a series of projections are acquired rather than the full spectrum.^105^ In this way, the analysis of the spectrum is facilitated as it consists of a collection of a series of 2D spectra. Automation of such analysis, called automated projection spectroscopy (APSY),^106^ yields a peak list of the full dimensionality spectrum without reconstructing it. The methods for ^13^C assignment purposes have been demonstrated with α-synuclein^107^ and successfully applied also for large intrinsically disordered proteins.^102^

### 2.3 CON and CACO Fingerprints

**Figure 6:**
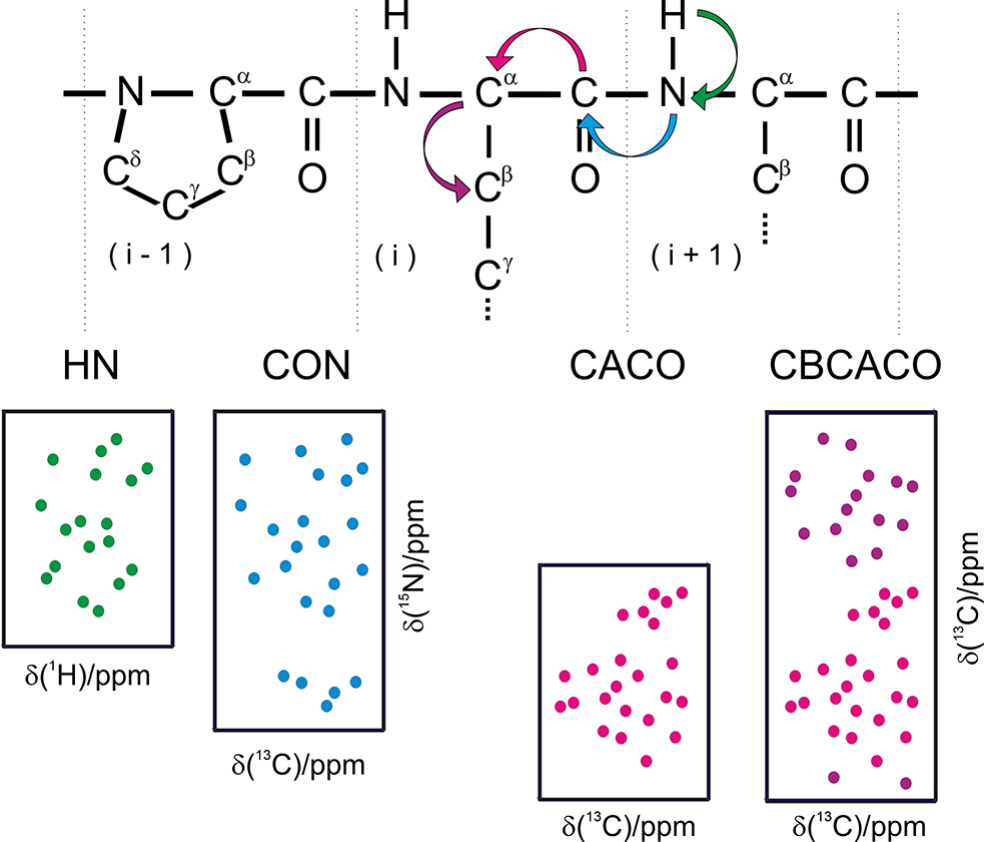
Schematic representation of the 2D spectra based on ^13^C direct detection that can be used, in conjunction with the 2D HN (^1^H–^15^N HSQC), to obtain a fingerprint of a protein. These include the 2D CON (^13^C′–^15^N correlation), 2D CACO (^13^C′–^13^C^α^ correlation), 2D CBCACO (^13^C′–^13^C^α^ and ^13^C′–^13^C^β^ correlations) spectra. The scalar couplings exploited to detect the correlations in the various spectra are schematically indicated in the top panel (^1^*J*_NH_, ^1^*J*_C′N_, ^1^*J*_C′C^α^_, ^1^*J*_C^α^C^β^_). In addition to the correlations involving backbone nuclei shown in the figure, correlations involving specific side-chains can be observed (for Asn and Gln in HN and CON 2D spectra and for Asn/Asp and Gln/Glu in CACO and CBCACO 2D spectra). Adapted from ref (112). Copyright 2014, Nature Publishing Group, a division of Macmillan Publishers Limited. All Rights Reserved.

Acquisition of 2D NMR spectra that provide well-resolved cross-peaks constitutes a useful tool to achieve a fingerprint of a protein. 2D HN spectra are often the first ones used for this purpose and sometimes the analogous 2D HC spectra are collected to obtain (and follow) ^13^C signals. However, information about carbonyl carbon nuclei is not available through ^1^H detected 2D spectra. It is thus worth to record experiments that correlate carbonyl carbon resonances with the directly bound heteronuclear spins, C^α^ and N, exploiting the one bond couplings (^1^*J*_C^α^C′_ and ^1^*J*_C′N_), to obtain the CACO and CON spectra. The various solutions for homonuclear decoupling described in section 1.2.1 ensure the removal of the large one-bond coupling in the direct acquisition dimension. The inclusion of an additional building block allows designing the 2D CBCACO and CCCO NMR experiments that collectively provide a suite of 2D NMR experiments that significantly enrich the information content that can be obtained through simple 2D experiments (Figure 6).^108^

The additional information available for a protein, if compared to that available in a 2D HN, is evident. The large dispersion of heteronuclear chemical shifts and additional nuclear spins that can be monitored (^13^C) provide a useful tool to investigate proteins in solution through simple 2D experiments. The correlation of nuclear spins belonging to two different amino acids involved in the peptide bond provides an important contribution to resolution in 2D spectra, particularly for intrinsically disordered proteins in which resonances tend to cluster in regions typical for each amino acids type.^69^ Interestingly, the protonless variants are amenable to combination with ^1^H detected NMR experiments using multiple receivers in order to collect two experiments simultaneously as shown through the CON//HN implementation.^109−111^

**Figure 7:**
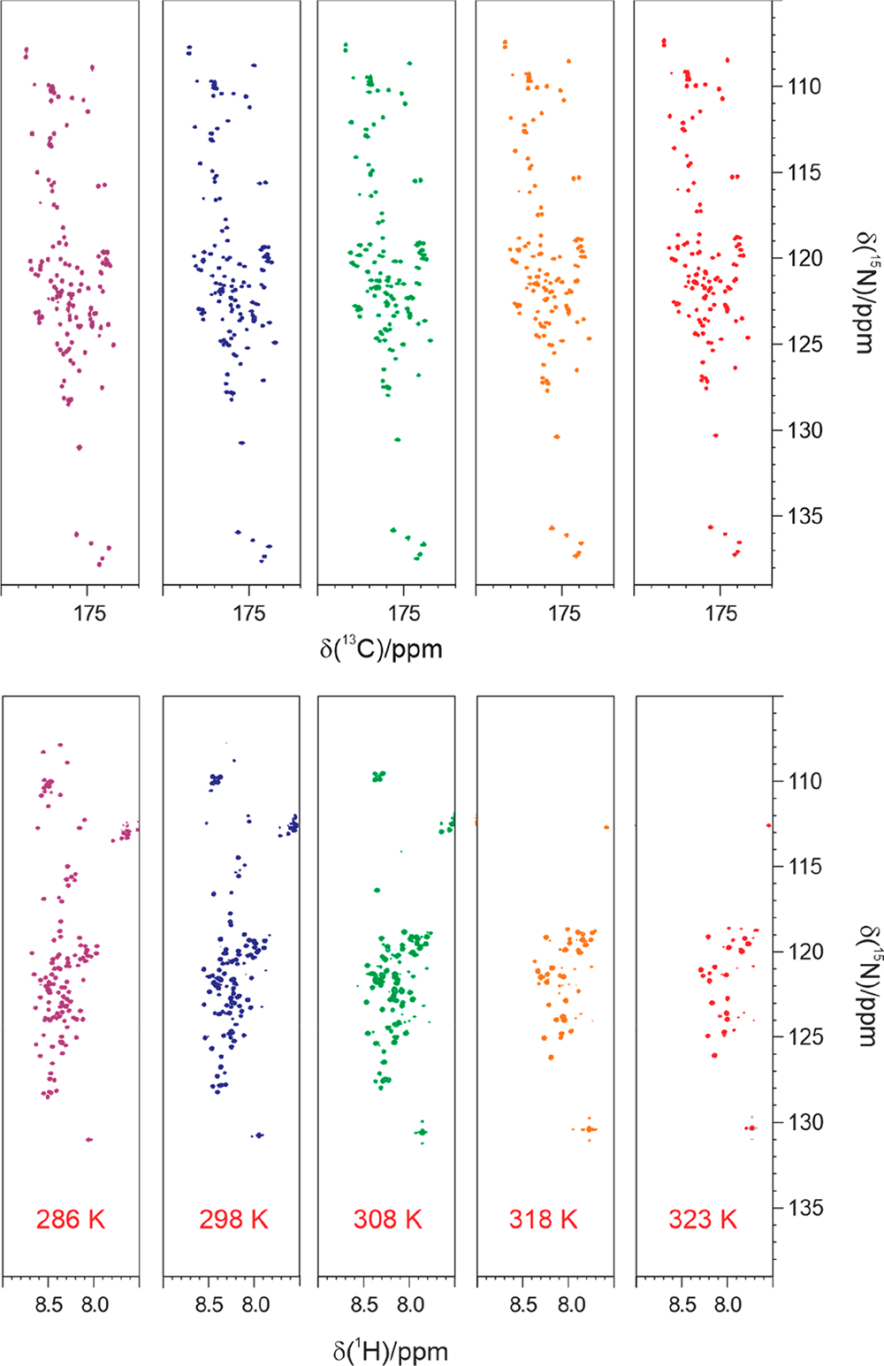
2D spectra correlating the backbone amide nitrogen either with the directly bound amide proton (^1^H–^15^N HSQC spectra, lower panels) or with the directly bound carbonyl (^13^C–^15^N CON spectra, upper panels) acquired on α-synuclein at pH 7.4, are shown as a function of increasing temperature, from 286 to 323 K (from left to right). Each spectrum was acquired at 16.4 T with one scan per increment on a 1 mM protein sample in 20 mM sodium phosphate buffer and 200 mM NaCl.

^13^C NMR is not particularly sensitive to solution conditions and is not affected by the detrimental effect of the presence of high salt concentration, pH, or temperature, which cause extensive line broadening of the H^N^ resonances in the NMR spectra.^8^ This holds in particular for the spectra of highly mobile proteins or protein regions, which have peptidic protons largely exposed to the solvent and not engaged in stabilizing interactions like H-bonds. In these cases, upon increasing experimental ionic strength, pH, or temperature, the quality of 2D HN correlation spectra may get worse due to the more efficient hydrogen-exchange mechanism, resulting in increasing number of peaks that are extensively line broadened, while the quality of ^13^C-detected spectra is maintained or improved. In Figure 7, the comparison of the 2D HN and 2D CON correlation spectra acquired for α-synuclein at pH 7.4 clearly shows that ^13^C NMR allows recovering the missing information in 2D HN spectra, such as the correlations of Gly, Ser and Thr residues, that are the first ones to disappear when increasing temperature.

As described in Section 1.2.2, amide protons can also provide a starting polarization source and different experimental variants of CON spectra have been proposed including ^1^H^N^-start,^27^^1^H^N-flip^,^59^^1^H^NBEST^,^63^ and ^1^H^N-CP^.^64^ These experiments lack correlations involving proline residues and the dependence on solvent exchange processes of amide protons is reintroduced that has an impact on the outcome of the spectra, eventually leading to loss of information when chemical exchange is very efficient. However, if properly tuned, this can even result in a sensitivity increase with respect to the ^1^H^N^-start variant by exploiting one of the variants with LRE (^1^H^N-flip^, ^1^H^NBEST^) or the ^1^H^N-CP^ one.

### 2.4 Useful NMR Observables

#### 2.4.1 Chemical Shifts and Heteronuclear Relaxation Rates

The process of sequence-specific resonance assignment provides a list of chemical shifts associated with different nuclei in the protein that represents the key to access atomic resolution information on complex macromolecules. These allow us to associate specific nuclei to each of the cross-peaks detected in multidimensional NMR spectra and follow spectral changes upon changes in the experimental conditions. For example, 2D spectra are often used to acquire protein fingerprints, as described in the previous section, and then follow spectral changes upon addition of potential partners, ligands, metal ions, etc. The additional contribution provided by ^13^C detected NMR experiments consists of the possibility to access information on solvent-exposed regions of proteins, such as external loops of globular proteins, intrinsically disordered proteins or protein regions, to follow proline residues and to recover information on paramagnetic proteins or highly deuterated large proteins.

**Figure 8:**
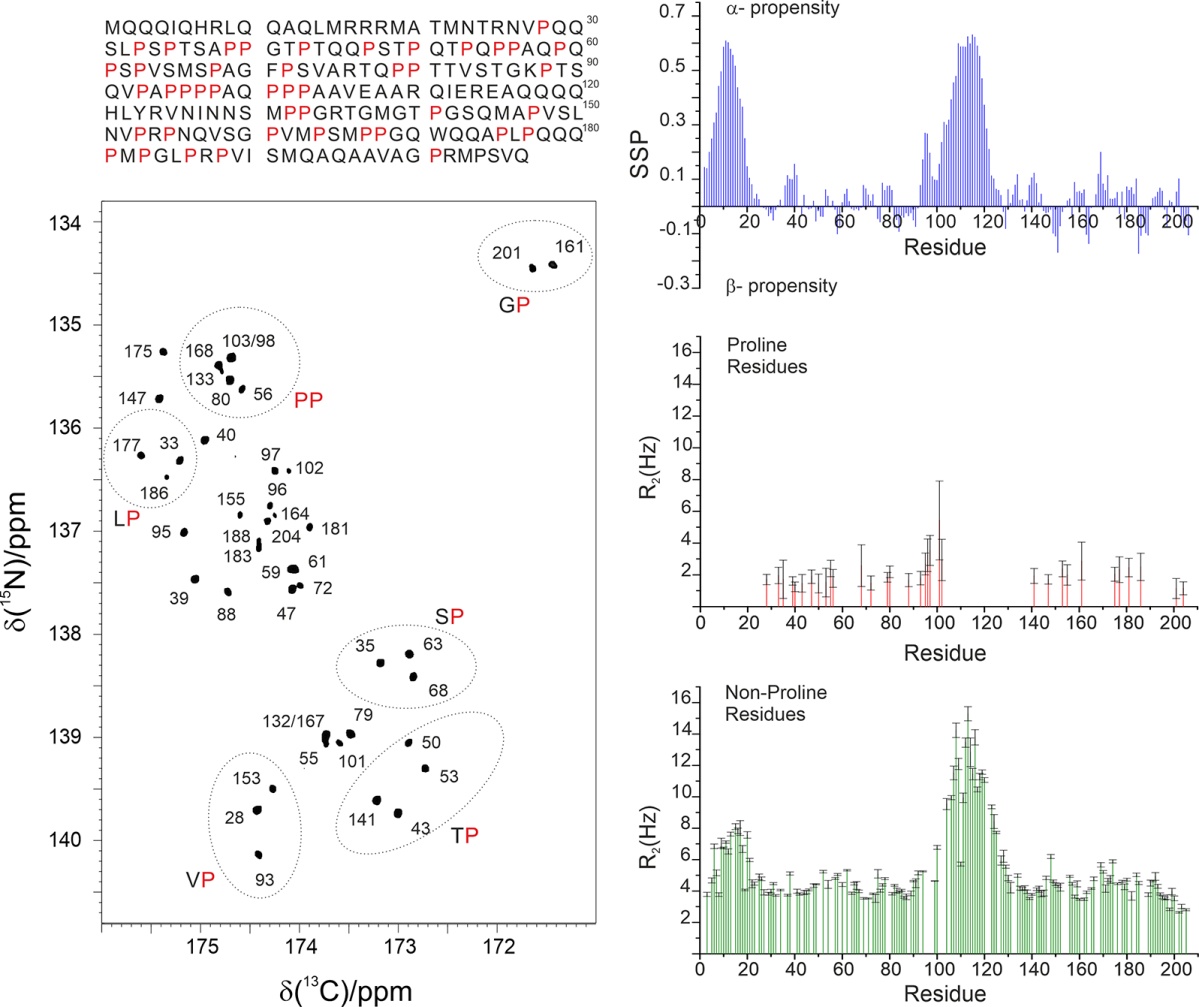
Left: 2D proline-fingerprint spectrum of CBP-ID4. In the 2D spectrum, the signals are numbered according to the proline position in the sequence; C′–N correlations involving identical residue pairs are circled. In the upper part, the primary sequence of the linker is reported, with the proline residues colored in red. Right: On the upper part, the secondary structure propensity (SSP) plot indicates that the protein is largely disordered, with two regions that have a measurable α-helical propensity. In the other two panels, ^15^N R_2_ data measured for proline nitrogen nuclei and ^15^N R_2_ data measured for the nonproline residues recorded at 16.4 T are reported. Adapted from ref (121). Copyright 2018 Wiley-VCH Verlag GmbH and Co. KGaA, Weinheim.

The chemical shifts, in particular heteronuclear ones, are also very informative about the secondary structural elements present in a protein.^113,114^ Indeed, on top of contributions deriving from the chemical structure and the presence of specific functional groups, a significant contribution also derives from the local conformation, which in turn is linked to the secondary structural element.^115,116^ Therefore, chemical shifts provide the first source of structural and dynamic information just by comparison with chemical shifts expected for a hypothetic “random-coil” state, representative of the contribution from the local chemical topology. The extraction of this information is by no means a trivial task. Computational tools have been developed to predict reference random-coil chemical shifts to enable the interpretation of experimentally determined chemical shifts in terms of structural and dynamic properties of a protein.^117−120^ As an example, Figure 8 shows the case of one of the “flexible linkers” of CBP, a 207 amino acids long intrinsically disordered region of the multidomain CBP protein (CBP-ID4).^99,121^ Even if largely unstructured, two partially populated helical elements separated by proline rich regions can clearly be identified from chemical shift analysis.

Variants of basic 2D experiments were developed to determine heteronuclear relaxation rates often used to access information about local flexibility.^122^ CON-based experimental variants are available to determine ^15^N relaxation rates (longitudinal and transverse).^123,124^ The most useful experiments however are probably those focusing on proline ^15^N spins as they provide information that is not accessible through ^1^H detected analogues. The possibility to focus on the ^15^N region of prolines selectively also allows researchers to focus on a narrow spectral region and achieve an excellent resolution with very few increments, if compared to the whole amide region in proteins, an attractive feature for the investigation of complex proline-rich proteins. It is interesting to note that the absence of the directly bound proton contributes to a reduction of the ^15^N nuclear relaxation rates, reflected in sharp NMR lines. As an example, the data obtained for CBP-ID4 are shown in Figure 8.

It is worth noting that the CON spectrum in Figure 8 reveals a peculiarity of this experiment when recorded for unstructured proteins, that is the clustering of the signals in groups depending on the previous amino acid type, whose nature determines to a great extent the chemical shift of the ^13^C′ nucleus, being all other contributions to the chemical shift typical of a folded protein largely averaged out.

With increasing pH and temperature, the efficient solvent exchange renders the interpretation of ^15^N relaxation rates for nonproline amino acids quite complicated as also exchange may contribute to the observed results, reducing possible advantages of CON-based approaches. In these cases, other heteronuclear relaxation rates involving nonexchangeable nuclei such as ^13^C may result useful and provide complementary information to that accessible through ^15^N relaxation. The picture is more complex because of the dense network of directly bound carbon nuclei, which implies ^13^C–^13^C interactions in uniformly labeled samples. However, cases of interest in which these effects are mitigated and valuable information can be obtained include the determination of carbonyl carbon longitudinal and transverse relaxation rates^123,125^ as well as heteronuclear ^1^H–^13^C NOEs.^126^ CON and CACO variants were used for the purpose and tested on model proteins;^123,127^ these may result useful for the investigation of intrinsically disordered proteins, for example, in all the cases in which chemical exchange is so pronounced to interfere with the determination of ^15^N relaxation rates.^128^

#### 2.4.2 Chemical Exchange

CON experiments also have interesting properties for the experimental determination of exchange processes with the solvent, an observable used since the early days of NMR to discriminate between amide protons easily accessible to the solvent from those protected into globular cores or involved in tight hydrogen bonds.^129^

In fact, the experiment correlates two heteronuclear spins not directly involved in chemical exchange with the solvent and it constitutes a useful tool to recover information also in conditions in which amide protons are broadened beyond detection, as described above (Figure 7).^63,72^ However, simple modifications of the basic pulse sequences can be designed to reintroduce a dependence on exchange processes with the solvent although in a more indirect way, allowing to monitor the process in a less perturbative way.

The ^1^H^N^CON variant^126,130^ of course constitutes the first obvious one to reintroduce effects deriving from solvent exchange through the starting polarization source used in the experimental variant.^112^ Exchange indeed influences the observability of the signals, even if to lower extent with respect to ^1^H detected experiments. Exchange is also responsible for a pronounced enhancement of the recovery of amide proton polarization to equilibrium provided amide protons are selectively perturbed with respect to those of water.^60,131,132^ Therefore, in a certain range of exchange rates, this effect can become a favorable feature for the detection of CON spectra as shown through the example of the ^1^H^NBEST^CON acquired on α-synuclein in less than 1 min^63^ (without introducing any delay between the end of one transient and the start of the next one thanks to the fast recovery of amide proton magnetization in the experimental conditions used). This is actually a qualitative observation of exchange which however could be quantified by exploiting similar approaches to those proposed by Schanda and Brutscher.^131^ Selective manipulation of the water resonance to highlight only residues whose amide proton senses this perturbation^133,134^ constitutes another widely used strategy that was implemented prior to ^1^H^N^-start CON variants to take advantage of the nice chemical shift dispersion typical of the CON reading scheme.^135^

However, probably the most interesting approaches are those that exploit the most simple CON variant, the ^13^C-start one, to indirectly detect effects of exchange by monitoring its effect through ^15^N spin coherences or spin orders. In an elegant paper, Atreya and co-workers proposed to monitor the effect of isotopic shift induced by deuterium in solutions constituted by 50% H_2_O and 50% D_2_O, and then measure exchange effects between the two resonances.^136^ Effects of chemical exchange can thus be determined by observing the direct exchange of polarization between the two isotopomers as well as by the effect on the scalar coupling of ^15^N with ^1^H, coupling that can be quenched by fast exchange processes. A similar approach was also proposed to investigate exchange rates of arginine side-chains.^137^

Finally, the CON reading scheme is particularly well suited to implement variants in which exchange is monitored through subtle effects on coherences or spin orders involving ^15^N. For example, the determination of ^15^N relaxation under CPMG, in the presence of efficient exchange processes of amide protons with the solvent, is influenced by whether ^1^H RF decoupling is applied or not. This difference, properly modeled, can be used to access information on exchange rates also in cases in which direct observation of the ^1^H resonance is precluded. This approach, initially designed to access information on exchange rates of selected amino acid side-chains^138−140^ was then implemented also for the study of amide protons in proteins in ^1^H and ^13^C detected experimental variants.^141−143^

Along similar lines, a three spin order operator can be created without perturbing the water resonance and then allowed to evolve in a free evolution period.^108^ In the presence of efficient solvent exchange processes, this becomes the major determinant of the disappearance of the three spin order by “decorrelation”, as initially described by Skrynnikov and Ernst.^144^ The CON variant allows implementation of this idea in a very clean way since the water resonance is not perturbed at all in the experiment and the 2D CON reading scheme allows to profit by the excellent resolution also in intrinsically disordered proteins.^108^

Finally, solvent accessible protein backbones can be “illuminated” by hyperpolarized HDO produced using dissolution-dynamic nuclear polarization (D-DNP).^145−149^ The hyperpolarized solvent provides a polarization reservoir, enhancing the ^1^H signals of sites undergoing chemical-exchange with the hyperpolarized solvent itself; implementation of a 2D CON reading scheme provides a nice resolution and reduced hurdles in dealing with the resonance of hyperpolarized HDO when performing ^1^H detection.^149^

#### 2.4.3 Residual Dipolar Couplings

Other observables deriving from dipole–dipole interactions include residual dipolar couplings (RDCs) resulting, as the term itself tells, from noncomplete averaging of dipolar interactions in solution.^150−152^ This can be originated by the natural magnetic anisotropy of the molecule under investigation that induces a partial degree of alignment when immersed in high magnetic fields or by external agents. Residual dipolar couplings are generally determined measuring changes in signals splitting resulting from a well resolved scalar coupling upon induction of a partial degree of alignment of the molecule in solution. The resolved ^13^C′–^13^C^α^ scalar couplings are thus an obvious candidate,^153^ easily accessible from carbonyl detected experiments, to determine RDCs. Experiments to measure a variety of different RDCs, including several that involve proton nuclei,^123,154,155^ were developed to enable the determination of RDCs also in cases in which methods based on ^1^H direct detection fails to provide information.^123,156^

#### 2.4.4 What about Homonuclear Nuclear Overhauser Effects (NOE)?

By analogy to observables that can be detected through ^1^H detected experiments, an important observable that comes in mind is the homonuclear NOE, one of the most robust sources of internuclear distances useful for solution structure determination. However, when moving from ^1^H to ^13^C, homonuclear NOEs are drastically reduced due to the lower gyromagnetic ratio of ^13^C.^157,158^ Ideally, long-range ^13^C–^13^C NOEs would be very useful observables that would contribute to the investigation of very large systems. However, since they scale with 1/*r*^6^, they become tiny effects that add to the much stronger interactions between directly bound carbon atoms (from 1 to 3 Å the NOE decreases by 3 orders of magnitude), so small that it is really difficult to measure them experimentally in uniformly labeled protein samples with the current hardware possibilities. On the other hand, ^13^C–^13^C NOEs can be easily detected between directly bound carbon nuclei and spin diffusion becomes a very efficient effect within these networks of directly bound carbon atoms.^9,19,30,52,158^ This information is not so useful to recover unknown structural information but contains nevertheless information that can be used for assignment purposes.

## 3 Biomolecular Applications

### 3.1 Focus on Amino Acid Side-Chains

The possibility to focus the NMR experiments on specific amino acids is a useful approach for proteins’ investigation. This can be used to aid the sequence specific assignment of macromolecules, as described in section 2.1, but it can also be exploited to address specific biological questions.

Amino acids’ side-chains are generally assigned using ^1^H-detected experiments, but there are many cases in which the resonances due to the nuclei of a long chain are missing or the signals vanish upon interaction due to conformational exchange. In all these cases, ^13^C-detected experiments can be of use. In addition, with simple 2D experiments is possible to focus on the key correlations relevant to monitor the behavior of a side chain upon changes in its environment, either due to changes in the solution conditions or to the interaction with a partner or to binding of a small molecule/metal ion. Since different kinds of nuclei are affected to a different extent by the various interactions, the analysis of carbon nuclei chemical shifts can provide additional information on conformational changes, variations in solvent exposure or in hydrogen bond pairing, etc. providing useful information to describe the processes of interest.

#### 3.1.1 Monitoring Side-Chains through C′-Detected Experiments

**Figure 9:**
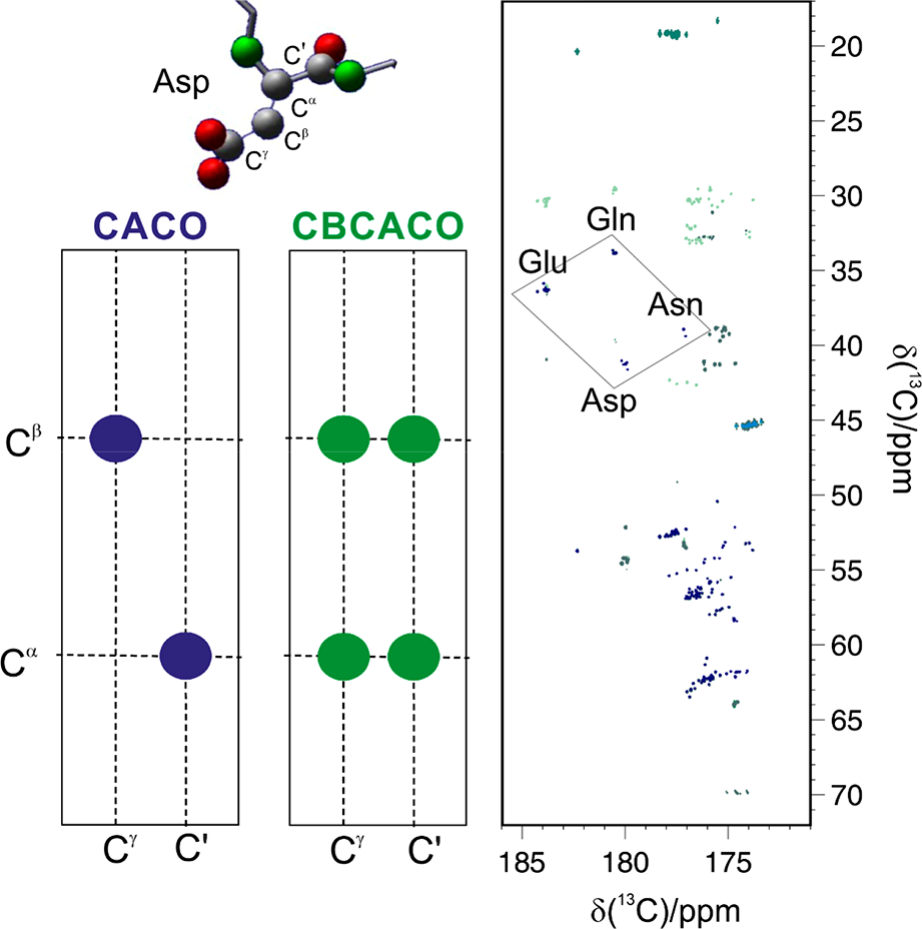
Superposition of the CACO (blue contours) and the CBCACO (green contours) spectra recorded on a 600 μM sample of α-synuclein in 20 mM Tris-Cl buffer, pH 7.4, and 310 K at 16.4 T. On the left, the correlations observable in the two spectra for an Asp residue, sketched above, are schematically reported.

The set composed of CACO, CBCACO, and CCCO experiments provides an excellent tool to monitor resonances of virtually every ^13^C nuclear spin of aliphatic side-chains by correlating them to the backbone carbonyl of each amino acid. Moreover, it also provides information on the terminal carbon spins of side-chains of residues that contain the carboxylate (aspartate and glutamate) or the carbonyl (asparagine and glutamine) moiety since their correlations can be detected in a clean region of the spectra. The one-bond correlation between the terminal carbon nuclei and the neighboring one can be collected in 2D CACO spectra; the cross-peaks observed for the side-chains of these four amino acid types fall in specific spectral regions, as illustrated in Figure 9. Comparison with a 2D CBCACO allows detection of the neighboring aliphatic carbon spin, providing the information to assign in a sequence-specific manner the ^13^C terminal resonance of aspartate and asparagine residues. The 2D CCCO is needed to complete the assignment and also identify in a sequence-specific manner the ^13^C resonances of glutamate and glutamine residues.

The CON experiment provides instead the intraresidue N^δ^–C^γ^ for asparagine and N^ε^–C^δ^ for glutamine residues. A combination of CON and CACO/CBCACO has been proposed to remove from the latter spectra the correlations due to the carbonyl carbon nuclei bound to nitrogen, producing maps where only the cross-peaks of aspartate and glutamate nuclei are present.^159^ Since the coupling between the backbone carbon and the carboxylate carbon of the aspartate side chain has a strong conformational dependence, in some favorable cases it is even possible to observe a splitting of the signals, whose magnitude can provide information on the chain’s conformation.

**Figure 10:**
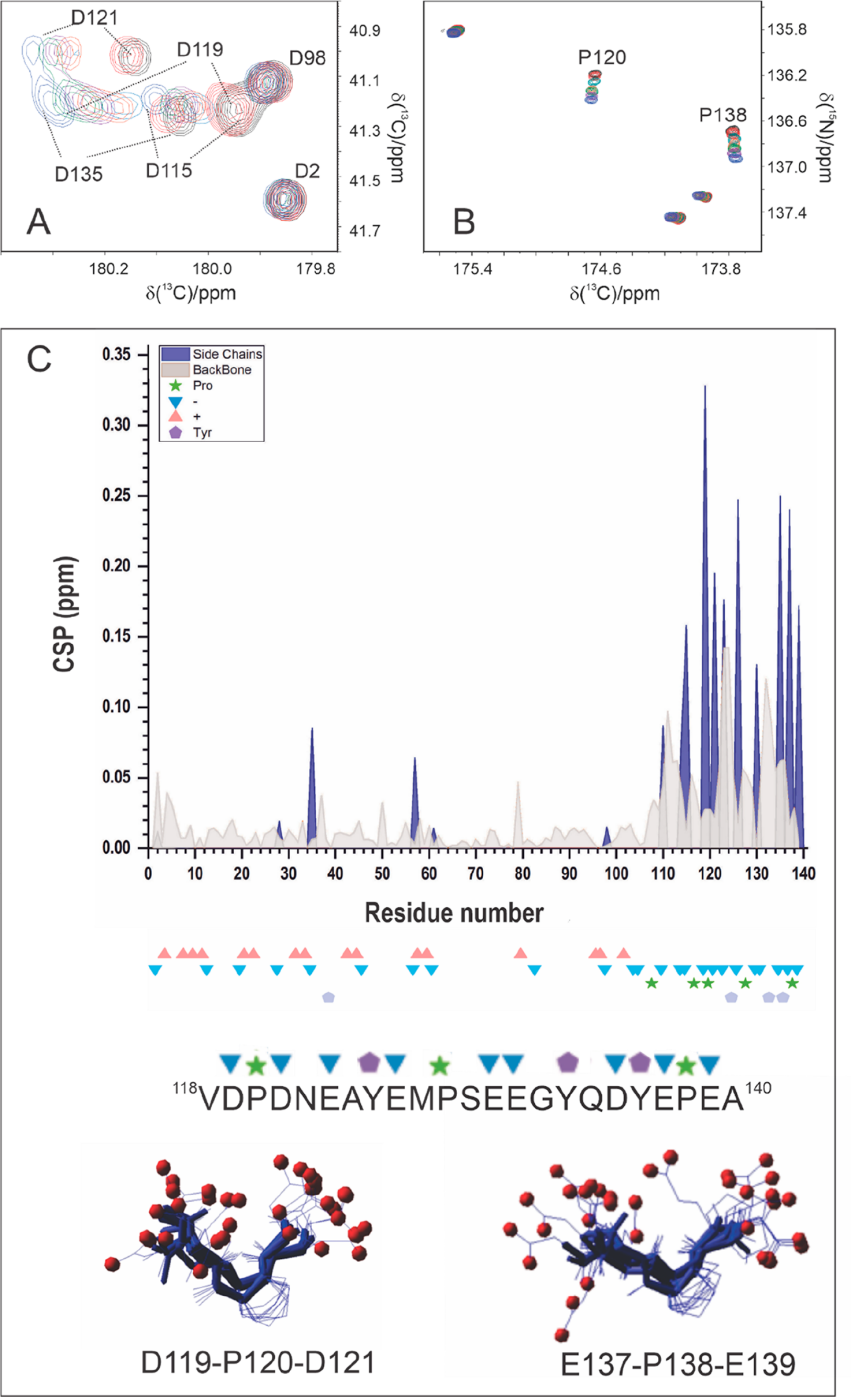
(A) Expansion of CBCACO spectra recorded on α-synuclein at increasing concentration of Ca^2+^ showing the chemical shift perturbation for the most affected Asp residues during the titration. (B) Expansion of CON spectra recorded on α-synuclein at increasing concentration of Ca^2+^ showing the chemical shift perturbation for two proline residues (P120 and P138). (C) Comparison of chemical shift perturbations (CSP) of side chain carboxylate/carbonyl carbon chemical shifts (blue) with backbone carbonyl carbon chemical shifts (red) determined through from 2D-CACO and 2D-CON spectra (CSP = |Δ(δ ^13^C)|). Backbone CSP values are smaller in magnitude with respect to those of side-chains and not necessarily maximal for Asp/Glu/Asn/Gln amino acids, reflecting a more indirect effect experienced by backbone nuclear spins upon interaction with calcium ions. On the bottom the portion of primary sequence affected by metal ion binding is reported, with two sketches of the three-amino acid motifs most affected. Adapted from ref (108). Copyright 2020 Wiley-VCH GmbH.

Carbonyl and carboxylate functional groups of amino acid side-chains are seldom assigned despite their key role in many biological processes and their investigation can be very instructive. A recent example in this context is provided by the investigation of α-synuclein when subject to concentration jumps of calcium metal ions, a process associated with the transmission of nervous signals. Negatively charged side-chains of aspartate and glutamate residues are expected to be among the first candidates to interact with positively charged ions such as Ca^2+^, and it is interesting to access direct information on these side-chain functional groups to identify whether specific regions of the polypeptide are affected to different extents by the interaction. The set of 2D ^13^C detected NMR experiments confirmed that the final part of the protein, rich in negatively charged amino acids, is the one sensing Ca^2+^ concentration increase, as also previously described through ^1^H detected experiments.^160−162^ These experiments, by monitoring also carboxylate groups of glutamate and aspartate residues, revealed that even within a disordered protein in which all of them are expected to be exposed to the solvent, only a subset of them are initially perturbed by the addition of Ca^2+^ (Figure 10A,C). Interestingly, among the most affected residues there are aspartate and glutamate residues separated by a proline, in a peculiar motif (DPD and EPE motif). The presence of a proline residue in between two residues featuring carboxylate groups could be a strategy to adapt the local conformation for Ca^2+^ interaction. It is noteworthy that these two proline residues were the most perturbed upon the addition of Ca^2+^, an observation that could be easily achieved through the 2D CON (Figure 10B). The 2D CACO and CON spectra thus provide a useful tool to focus on the metal ion coordination sphere and more generally to investigate interactions of negatively charged residues with complementary charged molecules in the cell.

#### 3.1.2 Positively Charged Amino Acids

Lysine and arginine side-chains are crucial for driving protein–protein interaction and for forming intramolecular and intermolecular salt bridges. Interactions and salt bridges often involve acidic protein side-chains such as those of aspartate or glutamate. Positively charged residues are relevant also to establish interactions with charged phosphodiester moieties of nucleic acids.

The first example of exploitation of CON and CBCACO type experiments for investigating protein–protein interaction dates back to 2006, in the study of the metal-mediated complex formed in the presence of Cu(I) between two copper chaperons, Atx1 and Ccc2a.^29^ These two proteins have a very specific role in copper trafficking and the solution structure of the complex obtained in the presence of Cu(I) was solved by NMR.^163^ However, the H^N^ signals of some of the residues considered important to characterize the metal-binding region were missing due to exchange with the solvent protons, preventing clear-cut characterization of their role. The correlations of these residues were instead present in the CON experiment and allowed the characterization of the interacting interfaces of the two oppositely charged proteins. In addition, through the comparative analysis of the CON and the CBCACO, it was possible to establish the direct involvement of selected carboxylate moieties in electrostatic interactions as well as in H-bonds necessary to stabilize the conformation of an otherwise very mobile region of the polypeptide.

Since Atx1 has several lysine residues considered important for driving binding, an experiment to detect the C^δ^–N^ε^ was designed, which allowed the identification of small but meaningful chemical shift variations upon complex formation allowing to delineate the interaction surface. One of the lysine residues (K65) experienced in the apo-form a very peculiar downfield shift of 6 ppm with respect to the usual value, suggesting its involvement in an intramolecular H-bond.^29^

Novel experiments were also designed for monitoring arginine side-chain. The guanidinium group of this amino acid is often involved in salt-bridges, H-bonds and in cation-π interactions with aromatic side-chains and with nucleic acid molecules. It is thus very informative to be able to determine its ionization state and its dynamics; however, ^1^H-detected experiments are often not effective due to line broadening and signal crowding and ^13^C detection has been exploited by several groups. For example, Hansen and co-workers proposed an experiment to obtain a ^13^C^ζ^–^15^N^ε^ correlation spectra avoiding transfer from ^13^C^ζ^ to ^15^N^η^. Such experiment proved useful for ^15^N relaxation measurements and quantification of the squared order parameter, S^2^, that reports on the motions of the arginine side-chain on some model proteins and on a 42 kDa enzyme, the human histone deacetylase 8 (HDAC8).^164^ With a similar approach it is possible to probe the rotational dynamics around the C^ζ^–N^ε^ bond.^165^ This experimental scheme found an interesting application on a mutant of T4 lysozyme, a 19 kDa protein containing 13 arginine residues with different exchange regimes: some are in fast exchange; five of them have ^15^N resolved resonances, suggesting some restriction in the rotation of the side-chain. A variant to include in the ^13^C detected scheme the so-called divided-evolution approach^166^ was designed and successfully demonstrated that two arginine residues have an exchange regime consistent with their involvement in a hydrogen-bonding network. An additional variant of the experiment including selective pulses proved useful to establish the exchange regime for two slower rotating guanidinium groups, which belong to residues shown to be involved in an interaction network together with a tryptophan side-chain in one case and in strong ionic bidentate hydrogen bonds in the other. Exploiting double-quantum experiments it is also possible to obtain ^13^C^ζ^–^15^N^ε^ correlation spectra with reduced exchange broadening, obtaining sharper lines. These can report about chemical shift perturbations occurring due to the interaction of the side-chain such as the measurement of very small isotopic effects when protons are substituted with deuterium to highlight the involvement of a specific group in stabilizing H-bonds or salt bridges.^167^

**Figure 11:**
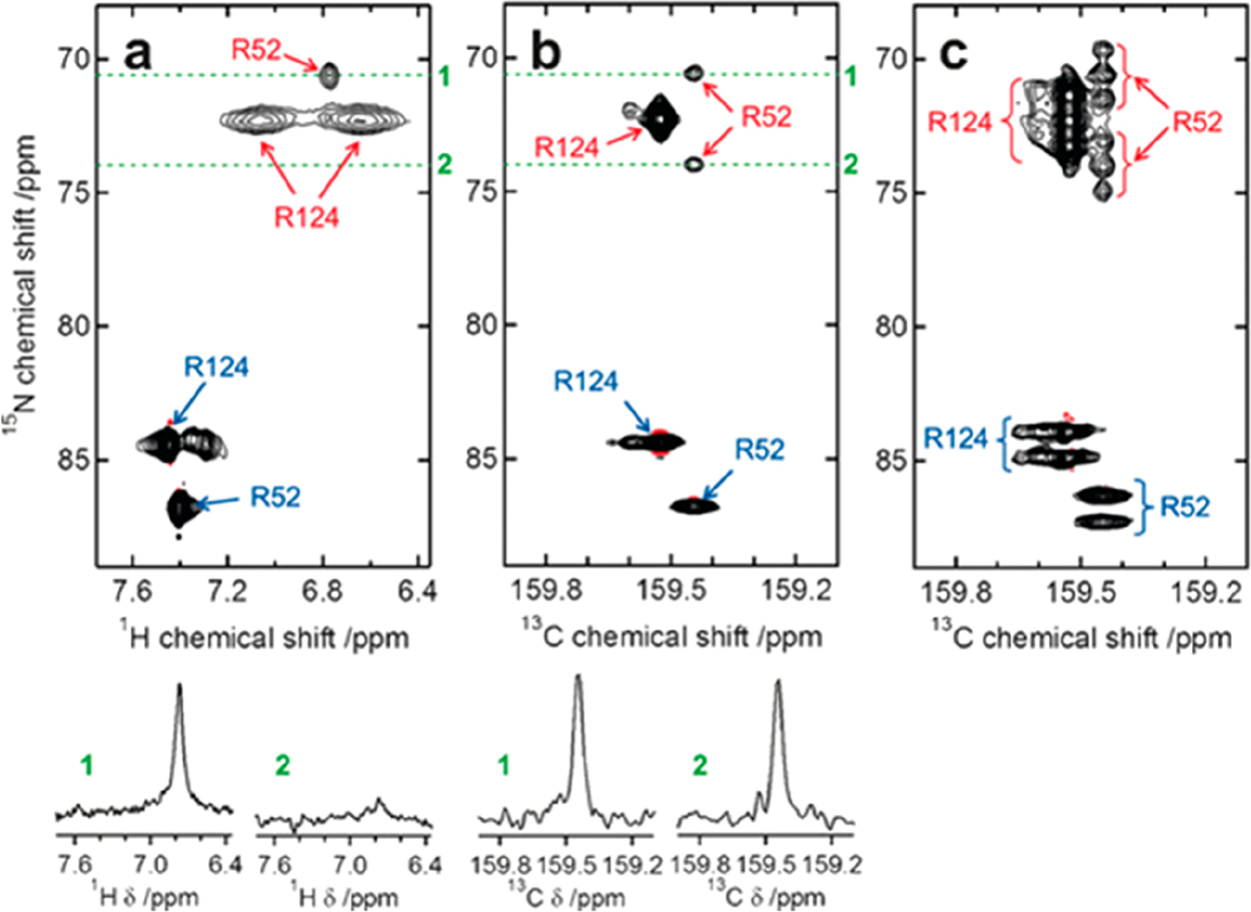
(a) ^1^H-^15^N HSQC spectrum of 1 mM PYP in 5 mM potassium phosphate at pH 6.2. The ^15^N^η^–^1^H^η^ and ^15^N^ϵ^–^1^H^ϵ^ correlations are indicated in red and blue, respectively. (b) ^1^H-decoupled and (c) ^1^H-coupled ^15^N^η/ϵ^–^13^C^ζ^ correlation spectra of 3 mM PYP in 5 mM potassium phosphate at pH 6.5. The ^15^N^η^–^13^C^ζ^ and ^15^N^ϵ^–^13^C^ζ^ correlations are indicated in red and blue, respectively. To avoid additional signals due to the presence of ^2^H isotopomers, D_2_O for field lock was added externally to a separate compartment of the NMR tube. The number of scans is as follows: (a) 16, (b) 128, and (c) 192. In (a) and (b), 1D traces from the 2D spectra, indicated by the broken lines, are shown. Reproduced with permission from ref (31). Copyright 2017 Wiley-VCH Verlag GmbH and Co. KGaA, Weinheim.

Another approach, proposed by Mulder and co-workers,^31^ provides the correlation of the ^13^C^ζ^ with both ^15^N^ε^ and ^15^N^η^, and enables the determination of the protonation pattern of the nitrogen nuclei. They tested the experiment, which based its efficiency on cross-polarization transfers, on three proteins differing in number of arginine residues present and in their protonation state. In the case of the photoactive yellow protein (PYP), which presents a particularly relevant arginine for protein function (R52), they demonstrated the possibility to detect all the protons bound to nitrogen nuclei, one H^ε^ and four H^η^, contrary to previous crystallographic studies (Figure 11).^31^

#### 3.1.3 Hydrophobic Amino Acids

The use of ^13^C direct detected NMR experiments has been proposed also to map methyl groups, often exploited for protein structure and dynamics, particularly in large proteins. In ^1^H detected experiments often the signals of the CH_3_ groups fall in regions where other signals resonate, complicating the extraction of selective information. A solution is to recur to selective labeling of the protein;^51^ otherwise, one can exploit a spectroscopic filter to remove all the nondesired cross-peaks, as in the ^13^C-Methyl COSY experiment proposed by Atreya and co-workers.^168^ In this experiment they exploited the fact that the ^13^CH_3_ signal of the methyl group located at the terminal end of an amino acid chain has only a single J-coupling to its directly attached ^13^C nucleus. Using this selection method they demonstrated that the ^13^CH_3_ correlation peaks for the various amino acids can be clearly separated into distinct spectral regions. Such a filtering building block can be incorporated in a 3D experiment wherein the additional dimensions can be utilized to provide intra-amino acid or long-range correlations. This approach is particularly well suited to study highly flexible proteins, for which the overlap is dramatic, but can be of use also for chemical shift mapping of large proteins or to obtain long-range information accurately quantifying paramagnetic relaxation enhancement.

Aromatic residues are often of great interest but not trivial to be investigated in detail, in particular in very large globular proteins or in intrinsically disordered ones. They often form the core of protein folds and recently they were shown to play a key role in the formation of membrane-less organelles through the process of liquid–liquid phase separation. The quaternary carbon (C^γ^) linking aromatic rings to C^β^ is not straightforward to be detected through ^1^H detected experiments while it gives rise to well resolved cross peaks with C^β^ that fall in a very clean spectral region of simple 2D ^13^C–^13^C COSY or TOCSY or NOESY spectra. These 2D spectra were used to identify metal ion ligands in paramagnetic proteins by comparison with the spectra obtained with a diamagnetic analogue.^44,45^ A special sequence was also designed to remove the two large one bond scalar couplings influencing the C^β^ (^1^*J*_C^β^C^γ^_ and ^1^*J*_C^β^C^α^_) to improve the resolution of 2D spectra that report the C^β^-C^γ^ correlation.^38^

#### 3.1.4 The Special Case of Proline

The cyclic nature of the proline side-chain and the lack of the backbone peptidic H^N^ atom sets it apart from all other natural amino acids. Well studied in globular proteins, proline residues are often very abundant in regions of proteins that would not lead to diffraction in X-ray crystallographic studies and on this basis were classified among “disorder promoting” amino acids in polypeptides. However, due to the constrained side-chain of proline residues, they confer local rigidity. The steric hindrance given by the cyclic structure of proline lowers the energy barrier present between the *cis* and the *trans* conformation of the peptide bond involving the nitrogen of this residue. When proline residues are part of globular folds the local structure can favor the *cis* conformation, still allowing interconversion to the *trans* conformer through minor changes, inducing a bend in the polypeptide and therefore protein folding.^169^ In highly flexible protein regions, while the occurrence of *cis* conformation is usually around 0.5% for all the residues in a polypeptide, for proline this can be much higher even in the absence of constraints from a stable 3D structure.^170,171^

The possibility to directly monitor correlations involving proline residues in clean regions of CON spectra stimulated the design of a series of experiments to investigate the properties of these residues in globular as well as disordered proteins. One of the first experiments proposed was the (H)CCCdNCO experiment,^82^ which correlates the intraresidue ^15^N and the ^13^C′ of the preceding residue with all the ^13^C resonances of a proline ring, providing information to identify the isomerization of the peptide bond. An alternative strategy to select in a clean way resonances involving proline nitrogen resonances consists of exploiting the evolution of the ^1^J_NH_ scalar coupling to suppress resonances of all amino acids except proline.^82^ Finally, when the ^15^N chemical shifts of prolines are sufficiently isolated from those of all other amino acids, such as for highly flexible disordered proteins (^15^N chemical shift range centered at 137 ppm), the use of a band selective pulse on ^15^N spins of proline residues allows to detect proline fingerprint with high resolution with a reduced spectral width and to design Pro-selective experiments to focus the assignment on proline residues. This experimental scheme allows tailoring all the experiments to measure observables such as ^15^N transverse and longitudinal relaxation rates.^121^ Lately, several other experiments were designed following a similar approach, which provides the sequence-specific assignment of proline residues to complement any assignment obtained either with ^13^C or ^1^H NMR (or, more likely, a combination of the two).^172^

**Figure 12:**
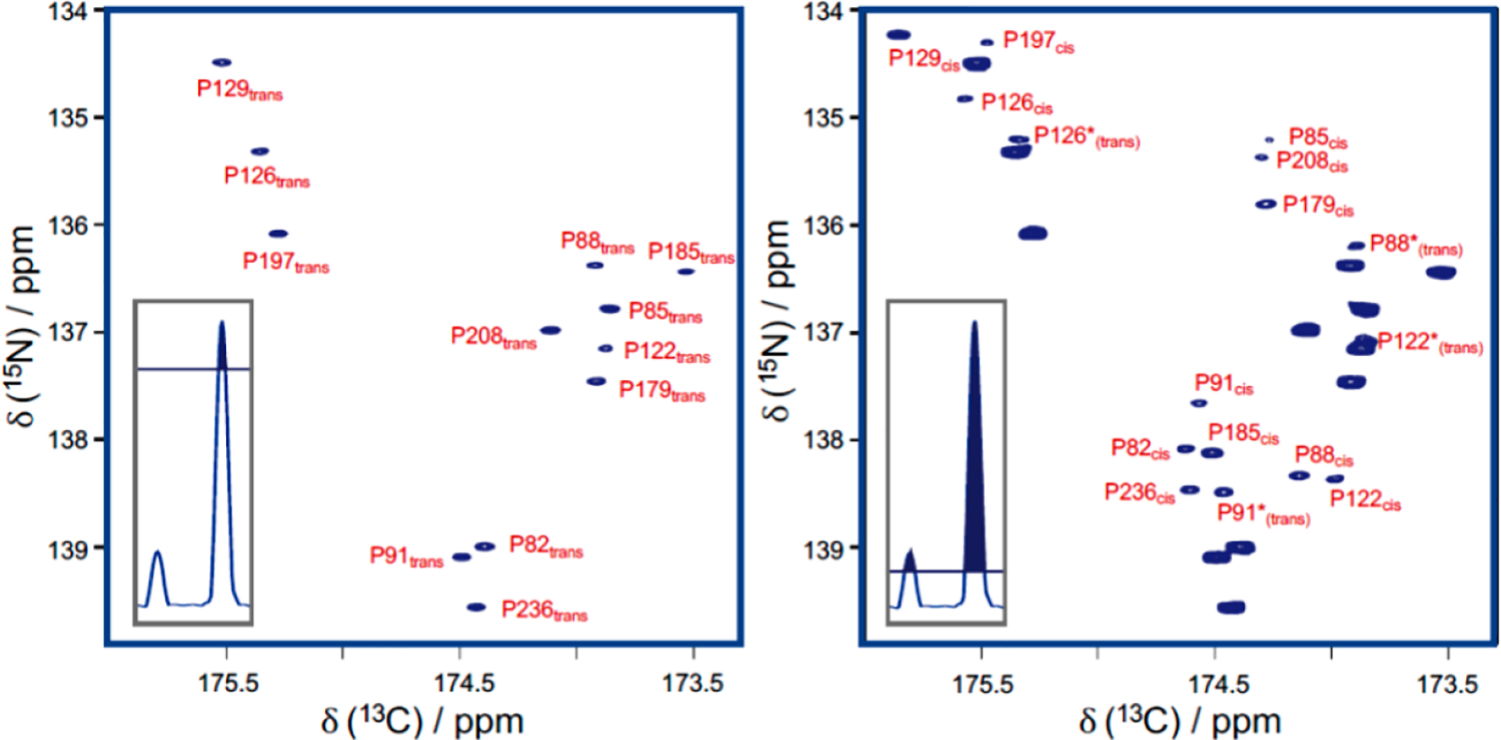
Proline region in the 2D CON spectrum. The main forms of prolines are observed (left) and minor forms appear (right) when the contour levels are lowered. In the inset, the level of the 2D slice and the relative intensity of major and minor forms are reported. Sequence specific assignment of cross-peaks is reported in the spectra; tentative assignments are indicated with an asterisk. Reprinted in part from ref (77). Copyright 2019 Elsevier Ltd. All rights reserved.

These experiments provided a wealth of information that is beginning to reveal possible functional roles of proline in highly flexible proteins. The investigation of flexible linkers of CBP, which constitute more than half of the primary sequence of this complex multidomain coactivator of transcription, revealed that often proline residues are clustered in specific regions of the primary sequence, indicating that these residues can be exploited to maintain elongation of the backbone, such as in the case of CBP-ID3,^101^ or act as helix-breakers, separating regions with high helical propensities, like in the case of CBP-ID4.^99^ Peculiar motifs with several proline residues in a row (PP, PPP, PPPP) could be detected and characterized with the novel experimental tools developed.^172^ Other motifs in which prolines are flanked by specific amino acids are also emerging, as described above for the interaction of α-synuclein with calcium ions through the DPD or EPE motifs. Similar motifs were also identified in different proteins, such as viral proteins^102,173−175^ as well as in WASP-interacting protein (WIP), an intrinsically disordered polypeptide with a key role in actin polymerization in activated T cells.^176,177^ Another interesting example in this context is provided by aromatic-proline pairs as recently monitored in different proteins^77,178,179^ and illustrated below through the example of quail osteopontin.^77^ The ^13^C detected experiments used to complete resonance assignment revealed in addition to the major form, also a subset of peaks with lower intensity that could be clearly identified in the clean region of CON spectra reporting the correlation of proline nitrogen (Figure 12). This set of peaks could be assigned in a sequence specific manner and the ^13^C chemical shifts of proline rings confirmed the presence of *cis*/*trans* isomers and provided their relative population. All major peaks have a secondary form and for some of them, which appear clustered in specific regions of the primary sequence, even additional forms due to various combinations of *cis* and *trans* conformation could be identified. The population of *cis* isomers results more abundant for proline residues near aromatic residues. In this protein the rigidity of the closed proline ring is exploited to establish π–π interactions with the aromatic side-chain of neighboring amino acids that imparts a compact state to the otherwise very flexible tertiary structure.^78^

An equilibrium between the *cis* and *trans* isomers of proline peptide bonds was shown to be relevant in case of RNA polymerase II C-terminal domain, an intrinsically disordered region of the protein.^178^ Another interesting observation was reported for the BMAL1 transactivation domain that is involved in circadian clock modulation,^179^ showing a direct link with function mediated by an aromatic-proline pair within an intrinsically disordered region. The ^13^C detected experiments are thus revealing information about novel sequence motifs that are not yet well described.

### 3.2 Overcoming Limitations Due to ^1^H Transverse Relaxation: Paramagnetic and Large Proteins

**Figure 13:**
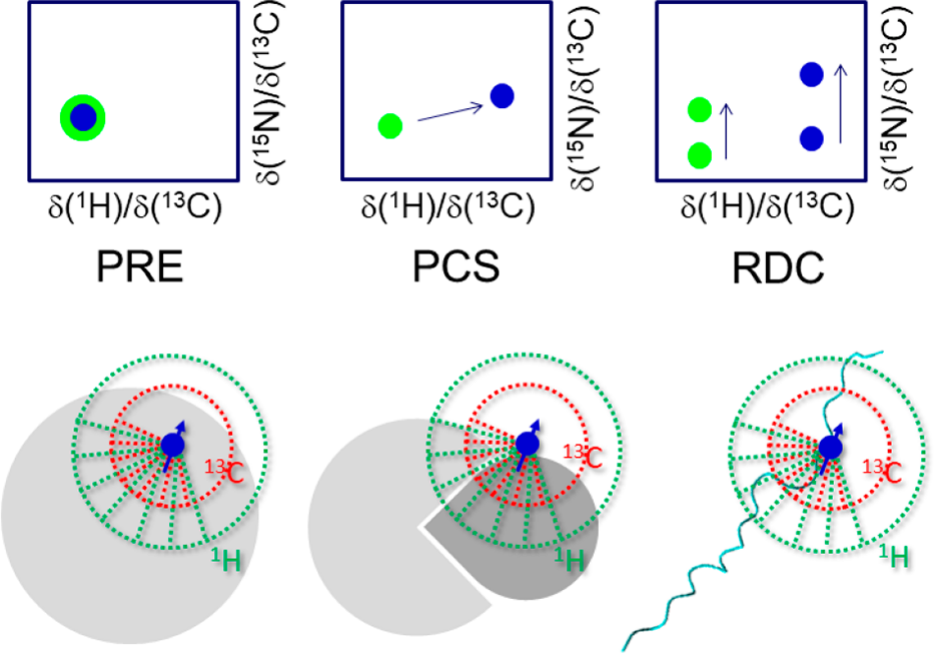
Presence of a paramagnetic center in a molecule affects its NMR spectrum (blue dots) with respect to the one of a diamagnetic analogue (green dots). The change in relaxation rates, in chemical shifts and signals splitting provide restraints called paramagnetic relaxation enhancement (PRE), preudocontact shift (PCS), or contact shift (CS) and paramagnetism-induced residual dipolar coupling (RDC), respectively. Paramagnetic effects on nuclear relaxation and chemical shifts are generally smaller for ^13^C with respect to ^1^H, and protonless NMR allows to measure observables closer to the paramagnetic center with respect to proton NMR. The approach can be applied for structural purposes to establish the reciprocal orientation of two interacting partners or to establish long-range crosstalk within a protein.

Paramagnetic centers are known to provide a wealth of additional contributions to nuclear spins in their surroundings, including contributions to nuclear relaxation, to chemical shifts as well as to partial orientation in high magnetic fields.^180^ The protonless NMR experiments provide, by default, additional unique information with respect to ^1^H detected experiments. They enable monitoring signals also at distances from the paramagnetic center in which the effects sensed by protons are so large that resonances are broadened beyond detection, recovering information that would be lost when using as reading schemes ^1^H detected experiments (such as ^1^H–^15^N or ^1^H–^13^C HSQCs).^43−47,181−185^ A variety of observables that contain structural information can thus be determined. Several approaches were proposed to quantify paramagnetic contributions to relaxation (either longitudinal or transverse, or both, depending on the type of paramagnetic center) and to exploit them to access distance information between nuclei and paramagnetic centers themselves for structure determination (Figure 13).^13^

Paramagnetic contributions to chemical shifts also contain a wealth of structural information and protonless NMR experiments enable detection of these effects also in regions of macromolecules in which protons are broadened beyond detection.^20,25,47,182,183^ The same holds for residual dipolar couplings that can be induced when a significant anisotropy derives from the paramagnetic metal ion itself.^123,156^ Therefore, the set of protonless experiments can provide excellent information for the structure determination of paramagnetic proteins.

After initial pioneering work showing the potential of the approach, the methods were applied to the study of type II Cu(II) containing proteins such as CopC^45^ as well as to oxidized Cu(II)Zn(II) superoxide dismutase^13^ to demonstrate that a 3D structure can be obtained also for proteins containing paramagnetic centers that broaden proton resonances beyond detection in large spheres around the metal ion itself. ^13^C detected experiments were also used to investigate binding of Cu(II) to αB-Crystallin^186^ as well as to follow the uptake of iron ions by ferritin.^187^ A similar strategy was used to investigate iron-containing proteins^182,188,189^ and calcium binding proteins where the metal ion was replaced with lanthanide ions.^47^ In the latter case, the lanthanide ion can be selected to modulate the magnitude and type of paramagnetic effects.^190^

Paramagnetic tags are also often engineered on purpose to study intermolecular interactions as well as possible residual structure in disordered proteins.^191−197^ The additional interactions deriving from the presence of a paramagnetic center are generally larger in magnitude with respect to internuclear ones on the grounds of the larger electronic magnetic moment. For this reason, they are well suited to reveal long-range information as well as information about short-range distances even if only partially populated in the ensemble of conformers describing disordered systems. In this context protonless NMR experiments provide valuable complementary information to that available through ^1^H detection.^198−200^ Actually, different variants of CON experiments were used to modulate the extent of paramagnetic effects that contribute to cross-peaks intensity by exploiting ^1^H-start variants (^1^H^α^, ^1^H^N^) and comparing them to effects observed through ^13^C-start variants, still exploiting the CON reading scheme to take advantage of its excellent resolution.^199^ Indeed while ^13^C-start variants only sense paramagnetic relaxation enhancements through ^13^C nuclei, ^1^H-start variants reintroduce a dependence also on effects sensed by protons, offering an additional way to modulate the extent to which paramagnetic effects are sensed.

**Figure 14:**
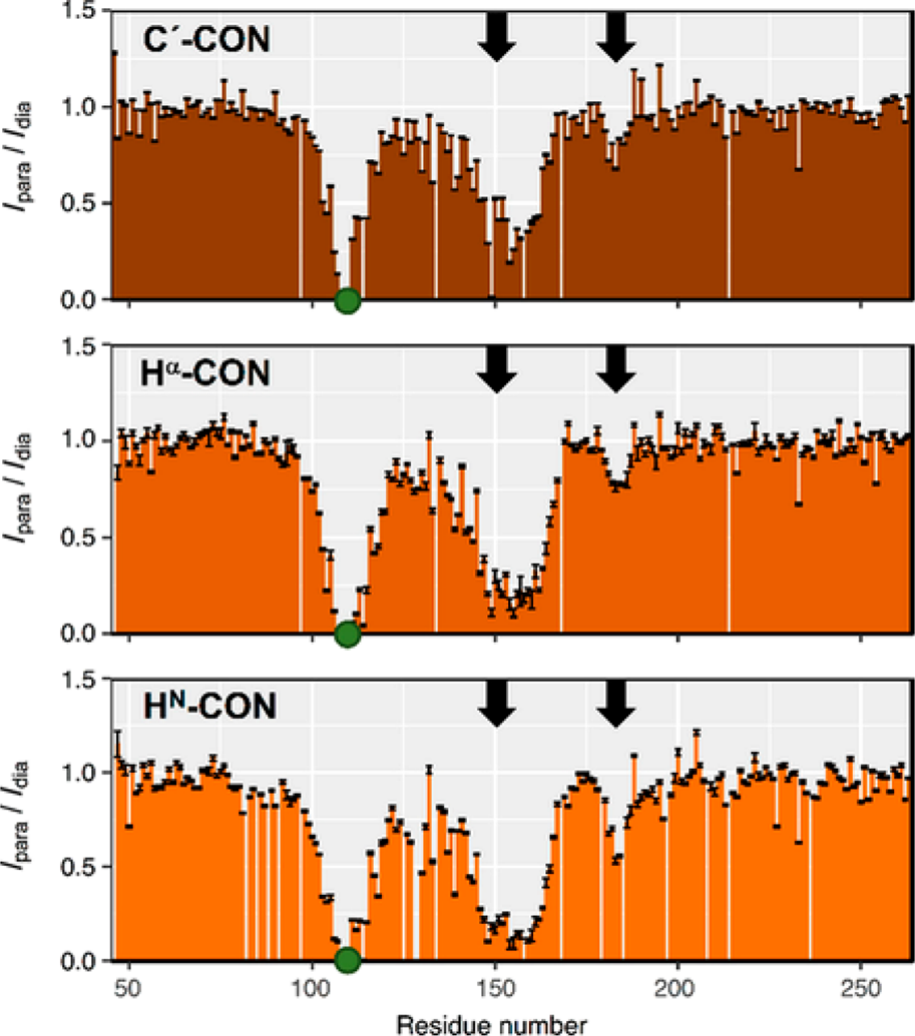
PRE intensity profiles determined through the CON-based approach exploiting different starting magnetization sources: C′-CON (top), H^α^-CON (middle), H^N^-CON (bottom). The green circle indicates the position of the spin label (S108C). Black arrows indicate the observed long-range contacts with the spin label. Reproduced with permission from ref (199). Copyright 2019 Wiley-VCH Verlag GmbH and Co. KGaA, Weinheim.

This approach was recently demonstrated for a highly flexible disordered system such as osteopontin.^199^ Upon comparing C′ and H^α^CON-derived long-range PREs in the protein grafted with MSTL, it appears that both experiments highlight the same regions of the protein (indicated by arrows in Figure 14), with the former showing less pronounced effects, in agreement with ^13^C nuclear spins sensing similar paramagnetic effects at shorter distances from the paramagnetic center with respect to protons. The comparison of the PREs observed through the H^α^ and H^N^CON, which both exploit ^1^H as a starting polarization source, highlighted the presence of proline residues, which are clustered in the vicinity of the so-called compact state of the polypeptide. In addition, the H^N^CON profiles are less uniform with respect to the H^α^CON profiles; a feature that could be due to additional solvent-mediated relaxation enhancement effects that are picked up by H^N^CON and do not influence H^α^CON data.

Paramagnetic tags can also provide useful long-range information about intermolecular interactions as well as about the relative orientation of two domains in multidomain proteins, such as for the case of tandem RNA Recognition Motif (RRM) domains (RRM1-RRM2) of the splicing factor U2AF65 bound to a polyuridine (U9) RNA oligonucleotide. A paramagnetic tag was introduced at position 155 in the RRM1 domain; the complementary information achieved through ^13^C protonless experiments in conjunction with ^1^H detected ones was very effective in defining the relative orientation of the two RRM domains connected by a flexible linker.^198^

Paramagnetic relaxation enhancements sensed by proteins upon addition of highly paramagnetic complexes to the solution (solvent PREs) are also modulated by the experimental tool used to measure them and protonless experiments resulted useful to access complementary information to that achieved through ^1^H–^15^N HSQC experiments.^201^ Alternatively, solvent PREs have been proposed as a tool to shorten longitudinal recovery times.^202,203^

Another area where heteronuclear direct detection can provide useful information on the grounds of the reduced contributions to relaxation sensed by heteronuclear spins with respect to protons as in case of paramagnetic proteins, is that of multimeric proteins^204^ or large protein assemblies for which extensive enrichment in ^2^H (in place of ^1^H) is needed to reduce the dipolar contributions to nuclear relaxation and thus the amount of protons that can be investigated is limited to exchangeable amide protons. These can be investigated through transverse relaxation optimized spectroscopy (TROSY)^205^ that exploits the constructive interference of CSA and DD interactions involving the amide proton and the directly bound amide nitrogen. A similar approach is more difficult to implement in a general way for ^13^C nuclear spins because of the higher variability in chemical topology and magnitude of the effects. Methyl TROSY,^206^ which exploits the interference between ^1^H–^1^H and ^1^H–^13^C DD interactions instead, represents the most successful application but only allows the study of methyl groups; other approaches have been proposed for aromatic rings as well as for methylene groups. However, on highly deuterated samples, ^13^C direct detection offers the most general strategy to recover information that is not easy to access otherwise. This approach, initially demonstrated on dimeric SOD (in the reduced, diamagnetic state)^9^ resulted useful to provide information on a multimeric protein of high molecular mass^52^ as well as for the Fc portion of immunoglobulin G (IgG) with a molecular mass of 56 kDa as model glycoprotein^207^ and for malate synthase G (MSG) protein (82-kDa).^32^ With increasing field strength, the utility of this approach is expected to increase thanks to the increased sensitivity and resolution provided by the increase in B_0_, in particular for aliphatic ^13^C resonances that only experience a modest contribution to relaxation due to the chemical shift anisotropy tensor at increasing magnetic field.^208,209^

### 3.3 Intrinsically Disordered Proteins

The field where ^13^C direct detection finds most applications is that of intrinsically disordered proteins (IDPs) or regions (IDRs) of complex multidomain/heterogeneous proteins. Research in this area flourished almost in parallel to the development of the suite of NMR experiments based on ^13^C direct detection, triggering the design of the most widely used experiments which in turn provided a wealth of valuable information at atomic resolution about their structural and dynamic properties. Since the early studies on unfolded systems, the importance of exploiting heteronuclei to increase the resolution of cross-peaks in multidimensional NMR spectra was recognized.^210^ Uniform isotopic labeling with ^13^C and ^15^N indeed contributed to the increase in the complexity of the systems that could be studied in unfolded states. However, initial studies were largely based on ^1^H direct detection, in particular on detection of amide protons that can be easily correlated to the directly bound ^15^N, the latter greatly contributing to excellent chemical shift dispersion, a key feature to overcome the main critical point arising from the high flexibility and lack of structure typical of IDPs. Amide protons however are influenced by exchange processes with the solvent (see section 2.4.2) and thus are prone to extensive line broadening in particular in conditions enhancing exchange processes. An interesting work^118^ analyzed the chemical shifts of disordered proteins deposited in the BMRB and revealed that most studies were based on ^1^H detected triple resonance experiments and were performed in conditions deviating from “physiological” ones because a reduction of pH or temperature (or of both) was necessary to diminish the impact of exchange processes on amide proton line widths and to obtain informative 2D HN spectra. In this context the possibility to shift to direct detection of ^13^C′ and exploit the suite of multidimensional NMR experiments offered an excellent alternative to investigate IDPs. Carbonyl carbon nuclei are not influenced by exchange processes and can be easily correlated to the directly bound nitrogen nuclei which, as mentioned above, are the nuclear spins that retain a large chemical shift dispersion also in the absence of a 3D structure. In addition the C′–N correlation exploits the scalar coupling deriving from the peptide bond, linking one amino acid with the neighboring one. This offers an important contribution to chemical shift dispersion deriving from the covalent structure of the protein which is particularly relevant for IDPs in which chemical shifts tend to average to those predicted for each amino acid type.^69^ This set of experiments was used to perform the sequence-specific assignment of a large number of IDPs, starting from initial work on α-synuclein,^23^ Src^211^ and securin.^75^ As a result, the size and complexity of IDPs investigated significantly increased, and assignments deposited were more extended as also proline resonances could be monitored contributing in this way to more accurate characterization of IDPs.

#### 3.3.1 Mimicking Physiological Events

The possibility to study IDPs in physiological conditions also represents an important motivation to choose ^13^C detected NMR, an important aspect in general but particularly for IDPs that are highly solvent-exposed and thus sensitive to environmental conditions.

Exposed backbones are often sites of post-translational modifications (PTMs), a field in which NMR can provide valuable contributions by highlighting at atomic resolution which amino acids are affected by these chemical reactions in real-time. Phosphorylation of OH groups of serine, threonine and tyrosine residues is one of the most extensively studied post-translational modifications through NMR.^212^ The impact of this reaction on amide protons and nitrogen chemical shifts is very large and 2D HN spectra have been often used to follow these reactions. However, several kinases change their activity depending on pH and temperature and it is often important to investigate their effect in conditions near physiological ones. Therefore, the possibility to follow these reactions with an additional experiment, the CON, in particular if acquired simultaneously to the 2D HN,^111^ offers an excellent tool of investigation for this challenging topic.^63,213^ Another strategy that has been proposed, in particular in cases in which sensitivity is an issue (either for low protein concentration or for instrumental setup) is the use of the most sensitive 2D experiment, the ^1^H-start CACO. Several variants focusing on glycine or nonglycine residues were indeed proposed and used to follow phosphorylation reactions, in the presence of purified kinases or in cell extracts, on a range of previously problematic targets, namely Mdm2, BRCA2, and Oct4.^203^

**Figure 15:**
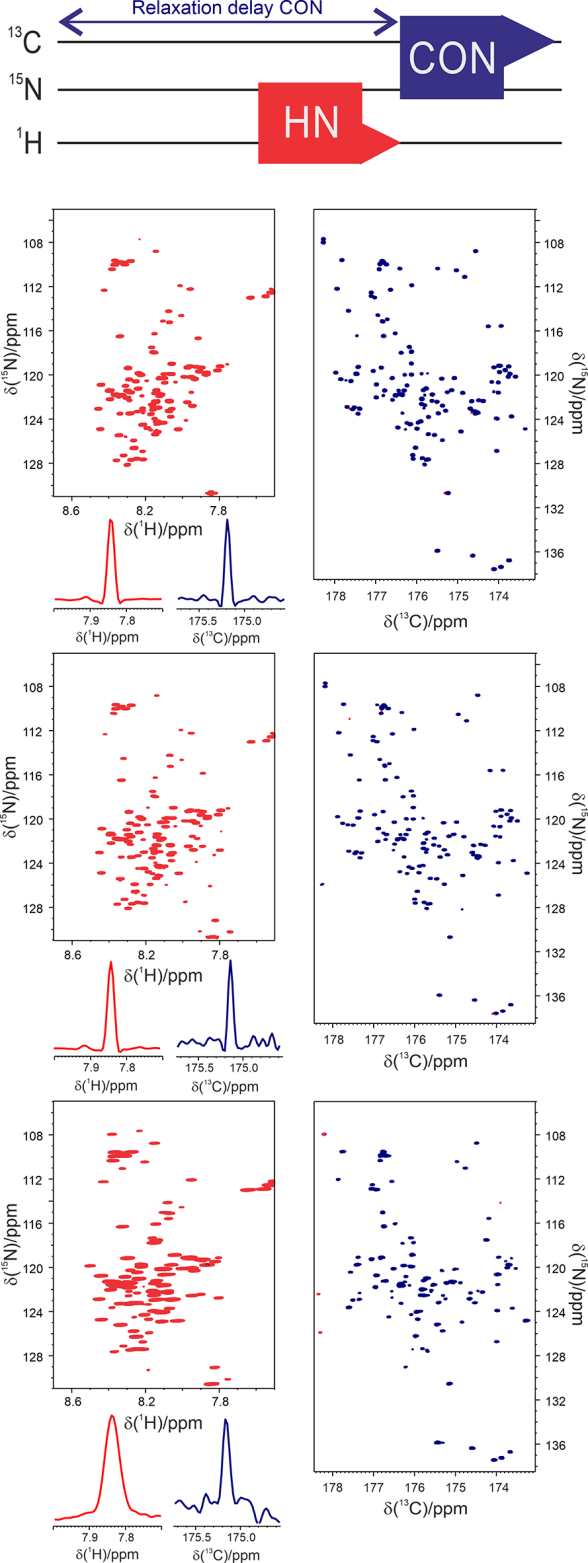
Scheme of the CON//HN experiment (top panel) and comparison of the 2D spectra (HN left, CON right) acquired through this experiment on ^13^C, ^15^N labeled α-synuclein at 310 K in different experimental conditions. From top to bottom: purified sample in 100 mM NaCl, 50 μM EDTA, 20 mM phosphate buffer at pH 7.4; in *Escherichia coli* cells lysate resuspended in the same buffer and in-cell. The traces of a representative signal extracted from the HN (red left) and the CON (blue right) spectra are also reported. Adapted with permission from ref (111). Copyright 2019 Biophysical Society.

In-cell NMR spectroscopy provides a unique spectroscopic tool to investigate a protein in conditions that resemble physiological ones and the potential of ^13^C detected experiments has been tested with several proteins characterized by different molecular mass and motional properties.^214^ The results clearly indicate that for well-folded proteins the method of choice is ^1^H NMR since line broadening typical for in-cell protein samples severely affects the detectability of the signals, particularly when CON-type experiments are used. The most valuable contribution provided by ^13^C detected experiments in this context consists of picking up in a clean way signals deriving from highly flexible protein regions that also retain these properties of high flexibility within whole cells. Indeed, when dealing with IDPs, ^13^C detected experiments offer a valuable solution to contrast the reduced chemical shift dispersion and high solvent exchange rates of the H^N^ signals, allowing to map entirely the protein backbone. The case of α-synuclein is paradigmatic.^112^ The complementary features of the different variants of 2D CON spectra (^1^H^N^-start, ^1^H^α^-start, ^1^H^α-flip^, etc.) as well as of CACO spectra (^1^H^α^-start, ^1^H^α-flip^) offer many opportunities to pick up the desired correlations within the lifetime of in-cell NMR samples. As an example, the H^NBEST^CON and H^α^ CON can be acquired in less than 1 h on in-cell α-synuclein samples.^63^ More recently, the CON//HN multiple receivers variant was implemented to monitor two experiments simultaneously,^111^ an important feature for samples of limited stability, such as in-cell NMR samples, showing that also the ^13^C-start variant of the CON can provide useful information in a very limited time (<1 h, Figure 15). Comparison of the two NMR spectra acquired simultaneously reveals that the increase in line width when passing to the in-cell samples is more pronounced for ^1^H than for ^13^C, a possible origin for this could be the local inhomogeneities that affect more nuclear spins with higher gyromagnetic ratios such as ^1^H. The simultaneous observation of the two experiments of course provides a wealth of atomic resolved information on the state of the protein within cells.

#### 3.3.2 Liquid–Liquid Phase Separation

Liquid–liquid phase separation (LLPS) is a phase transition process in which a homogeneous solution separates into two different coexisting liquid phases. The interface between the dense and the light phases allows the passage only of certain molecules making these droplets act as compartments which are called membrane-less organelles.^215^ IDPs and protein containing IDRs are over-represented among proteins that undergo phase separation processes, especially in condensates containing RNA. This is likely due to their ability to form transient multivalent interactions. The LLPS behavior of IDPs is governed by various interactions, going from charge–charge interactions to π–π and cation−π interactions as well as hydrophobic contacts and charge pairing; in vitro, phase separation is often driven by changes in pH or temperature or ionic strength of the solution.^216^

In living cells, condensates can act as organizational centers promoting reactions or sequestering proteins and nucleic acids to inhibit metabolic pathways and it is crucial to be able to understand the driving forces leading to their formation. Furthermore, phase transitions are also associated with a range of neurodegenerative diseases and it is pharmaceutically relevant to understand how to interfere with their formation.^217,218^ NMR spectroscopy is one of the main players on this grounds and ^13^C NMR has been exploited to characterize at the molecular level some of these processes.

**Figure 16:**
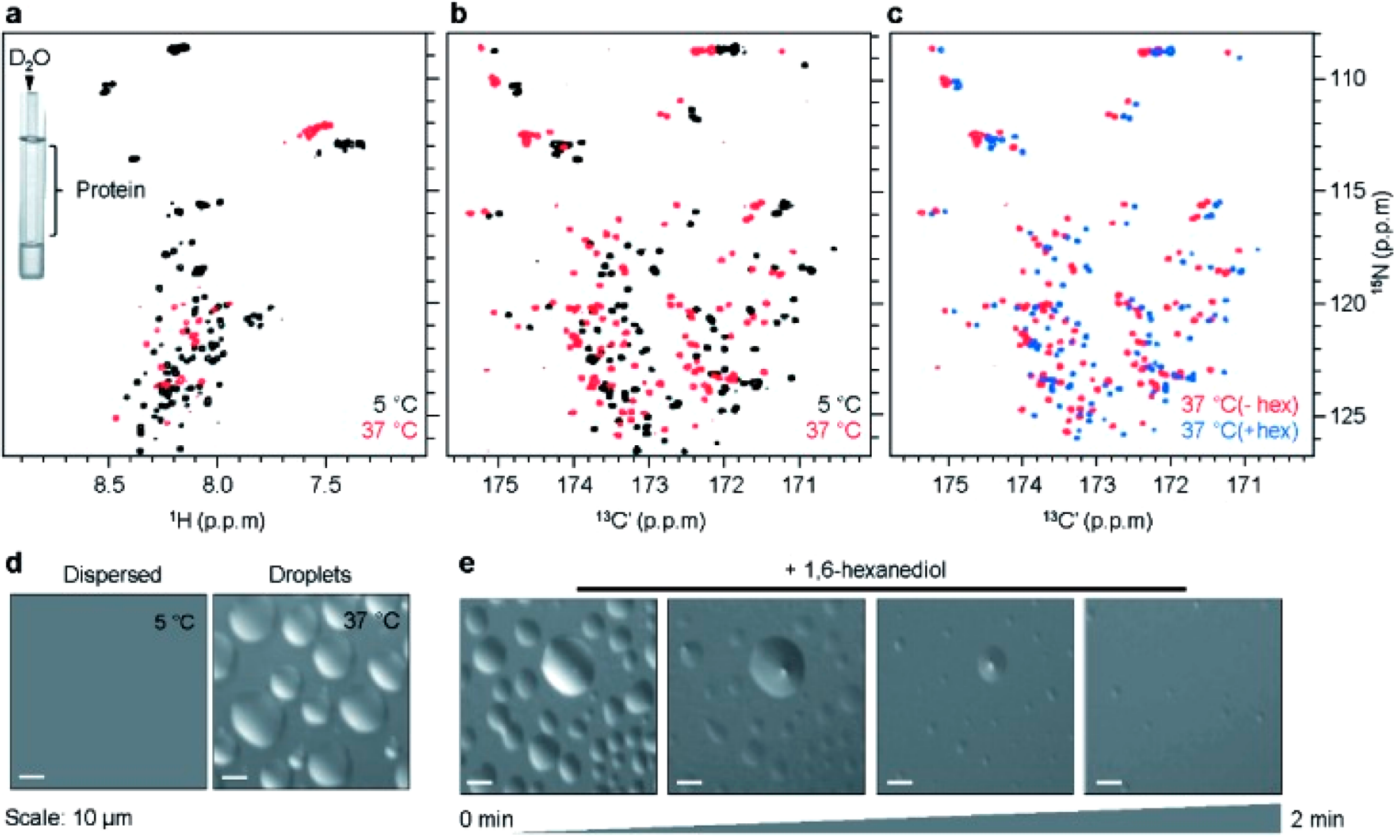
NMR spectroscopy of liquid–liquid phase separation of the repeat domain of tau. (a, b) Superposition of 2D ^1^H-^15^N HSQC (a) and 2D CON (b) spectra of K18 in the monomeric dispersed state (5 °C, black) and the droplet phase (37 °C, red). To avoid contributions of solvent exchange to NMR signal broadening in ^1^H-^15^N correlation spectra, D_2_O was placed into a separate capillary tube (insert in a). (c) Superposition of CON spectra of K18 recorded at 37 °C in the absence (−hex, red) and presence (+hex, blue) of 3% 1,6-hexanediol, which rapidly dissolved K18 droplets (e). (d) DIC microscopy demonstrates K18 droplet formation at 37 °C. (e) Time-dependent dissolution of K18 droplets in the presence of 3% 1,6-hexanediol observed by DIC microscopy. Reproduced from ref (220) with permission from the Royal Society of Chemistry.

An interesting example is the phase transition of Tau, an IDP that functions in microtubule nucleation, assembly, and stabilization. This large protein contains a N-terminal projection domain, two proline-rich regions, and a C-terminal domain that includes three (3R) or four (4R) imperfect 31- or 32-residue repeats. The repeat domain constitutes the microtubule-binding domain and has the ability to bind microtubules and to promote their assembly.^219^ Tau and its shorter variant containing the 4R repeats (termed K18) undergo liquid–liquid phase separation in vitro at pH 8.8 and 37 °C (Figure 16) and ^13^C NMR was key to be able to map at the residue level changes occurring upon LLPS formation since most of the signals of ^1^H^N^-detected spectra, and those of Gly, Ser and Thr residues in particular, were broadened beyond detection in these conditions. The high quality of the spectra obtained, including CON and CBCACO, suggested the possibility to follow chemical shift perturbations for each residue and highlight changes in secondary structure propensities upon droplets formation.^220^

Another example was recently provided by the investigation of the C-terminal disordered regions of two interacting proteins, FMRP and CAPRIN1.^221^ These two fragments form condensates individually and in interaction when FMRP is phosphorylated in specific positions. The comparison of the CON spectrum of CAPRIN1 in the presence of phase-separated FMRP and of CAPRIN1 cophase-separated with phosphorylated FMRP (pFMRP) highlighted the involvement of two arginine-rich regions in the interaction as well as the involvement of some tyrosine residues.

#### 3.3.3 Low Complexity Regions

“Low complexity regions” is a term widely used to describe parts of polypeptide chains constituted by a simple composition in terms of amino acids, featuring a few or even only one amino acid-type. These are not expected to be parts of globular folds, at least considering what we know as of today, and are often found as parts of IDPs/IDRs.

**Figure 17:**
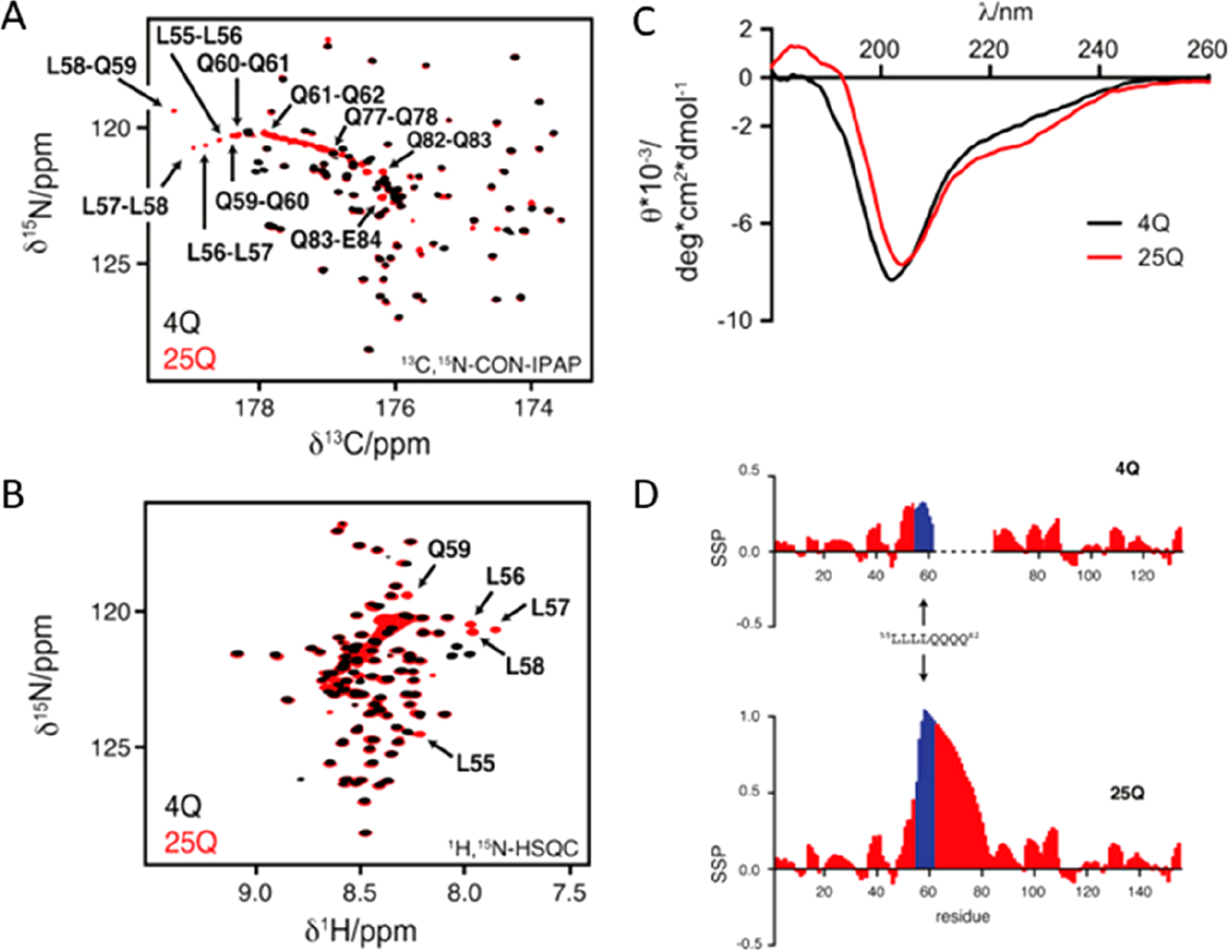
Comparison of the (A, B) NMR and (C) CD spectra of 4Q and 25Q; secondary structure propensity of 4Q and 25Q is reported in (D). The central region of (A) the ^13^C,^15^N-CON-IPAP spectrum and (B) the ^1^H–^15^N HSQC spectrum of 400 μM 4Q (black) and 25Q (red) at 278 K are shown, with the assignment of the resonances that experience the largest chemical shift variations upon increasing the length of the poly-Q tract. (C) CD spectrum of 120 μM 4Q and 130 μM 25Q at 310 K. In (D) Values for residues 55 to 62, corresponding to the ^55^LLLL^58^ motif and the first 4 Gln of the poly-Q tract, are shown in blue to highlight the variation of the structural properties of the protein due to the different length of the poly-Q tract. To facilitate the comparison, the values for the residues of 4Q that follow the poly-Q tract are shifted to the right by 21 units. Adapted from ref (100). Copyright 2016 Biophysical Society.

Among them an interesting example is provided by the so-called poly-Q, that is parts of polypeptide chains constituted by many glutamine residues (Qs) in a row. These motifs attracted the attention of the scientific community because some proteins featuring elongated stretches of Qs are linked to the onset of the so-called poly-Q diseases, a set of neurodegenerative diseases caused by malfunction of proteins that only have in common a part of the polypeptide chain with a high number of glutamine residues.^222^ The atomic resolution structural and dynamic investigation of these proteins is not easy because they do not crystallize, preventing the use of X-ray crystallography, and also from the NMR point of view their investigation represents a challenge due to the highly repetitive primary sequence. With ^13^C detected NMR experiments it was possible to perform complete sequence specific assignment of the N-terminal fragment of the androgen receptor (AR) comprising up to 25 Q in a row (Figure 17), not just as an isolated peptide but embedded in the N-terminal fragment of AR (135 and 156 amino acids in the 4Q and 25Q variants, respectively). This study revealed a pronounced helical propensity of this region and highlighted the important role of four leucine residues preceding the poly-Q tract to induce helicity in the poly-Q region, as confirmed through a deletion mutant.^100,223^ This may provide a mechanism to protect the poly-Q tract from aggregation, a relevant process for the onset of a neurodegenerative disease (spinal bulbar muscular atrophy). Interestingly, these poly-Q helices, induced by preceding leucine residues were also identified in functional proteins such as the ID5 linker of CBP.^224^ The investigation of poly-Q containing proteins has now become a very active field of investigation.^225−227^

## 4 Conclusions and Outlook

Largely neglected for many years in favor of approaches exploiting direct detection of proton on the grounds of the intrinsic higher sensitivity of the latter, from the early 2000s ^13^C direct detected NMR in solution has been rediscovered thanks to hardware development and experimental improvements.

The first spectra we recorded at 16.4 T (700 MHz ^1^H frequency) were obtained with a spectrometer equipped with a room-temperature inverse detection probehead, with a S/N ratio on the ASTM standard sample of about 200:1; today we are routinely using a cryogenically cooled probe for direct ^13^C observation with a S/N ratio of 2800:1 on the same sample. This increase in sensitivity opened the way to routine use of ^13^C direct detection by allowing a significant reduction of experimental times to access the same information (a factor of about 200). With the latest magnet and probeheads technology, the instrumental sensitivity jumps above 4400 for a 28.1 T (1.2 GHz) spectrometer.

In a stepwise fashion, solutions to overcome the potential limitations of the approach have been devised. Automation for removing the large splitting due to one bond scalar couplings in the ^13^C direct dimension constituted an important contribution for making it handy. Different polarization sources were used as the basis to design a wide variety of NMR experiments, ranging from simple 2D experiments to achieve a snapshot of a protein in solution, to 3D experiments for sequence-specific resonance assignment, all the way to complex multidimensional experiments (nD, with *n* > 3) exploiting the most advanced strategies to acquire and process the data. In addition, a wide variety of additional experiments to detect different NMR observables were developed and made available in the NMR spectrometers’ library of pulse sequences, thus becoming of general use.

Browsing the literature, it can be realized that next to papers dedicated to the development of the experiments and exploring potential novel applications, there are many other papers including ^13^C detected experiments side by side to ^1^H detected ones for fingerprinting or to complete the characterization or the assignment of a protein. This is a clear indication that since the early 2000s when it was considered a sort of revival of a vintage technology, ^13^C direct detection NMR in solution is now considered a useful complement to ^1^H NMR that can provide unique information or simplify assignment and characterization of biomolecules in general.

One of the research areas where ^13^C direct detection has proven very successful, as also witnessed but the many applications populating this account, is the characterization of intrinsically disordered proteins and heterogeneous proteins presenting highly mobile portions of their chain. Indeed, ^13^C NMR provides larger chemical shift dispersion with respect to proton NMR and benefits from the favorable relaxation properties of disordered polypeptides. We expect that this area will be even more important in the future since these highly flexible disordered proteins constitute a large share of the proteome and need to be investigated at atomic level.

This leads to the topic of the capability of highly flexible proteins to engage in intermolecular interactions still in a nonspecific way, interactions that lead to fuzzy complexes as well as to the process of liquid–liquid phase separation, still not well characterized. The investigation of these interactions at atomic resolution has great potential to reveal the driving forces of these processes and exclusively heteronuclear experiments can play a central role, monitoring both protein backbone as well as key side-chains involved in this process.

## Abbreviations

AP: antiphase
APSY: automated projection spectroscopy
BMRB: biological magnetic resonance data bank
CPMG: Carr–Purcell–Meiboom–Gill
CS: compressed sensing
CSA: chemical shift anisotropy
DD: dipole–dipole
D-DNP: dissolution dynamic nuclear polarization
DIPAP: double IPAP
DS3E: double S3E
FFT: fast Fourier transform
FID: free induction decay
INEPT: insensitive nuclei enhanced by polarization transfer
IP: in-phase
LRE: longitudinal relaxation enhancement
MDD: multidimensional decomposition
ME: maximum entropy
MFT: multidimensional Fourier transform
NMR: nuclear magnetic resonance
NUS: nonuniform sampling
PCS: pseudocontact shift
PRE: paramagnetic relaxation enhancement
PTM: post-translational modification
RDC: residual dipole coupling
S3E: spin states selective
SMFT: sparse multidimensional Fourier transform
